# Catalytic mechanism and molecular engineering of quinolone biosynthesis in dioxygenase AsqJ

**DOI:** 10.1038/s41467-018-03442-2

**Published:** 2018-03-21

**Authors:** Sophie L. Mader, Alois Bräuer, Michael Groll, Ville R. I. Kaila

**Affiliations:** Center for Integrated Protein Science Munich (CIPSM), Department Chemie, Technische Universität München, Lichtenbergstraße 4, 85748 Garching, Germany

## Abstract

The recently discovered Fe^II^/α-ketoglutarate-dependent dioxygenase AsqJ from *Aspergillus nidulans* stereoselectively catalyzes a multistep synthesis of quinolone alkaloids, natural products with significant biomedical applications. To probe molecular mechanisms of this elusive catalytic process, we combine here multi-scale quantum and classical molecular simulations with X-ray crystallography, and in vitro biochemical activity studies. We discover that methylation of the substrate is essential for the activity of AsqJ, establishing molecular strain that fine-tunes π-stacking interactions within the active site. To rationally engineer AsqJ for modified substrates, we amplify dispersive interactions within the active site. We demonstrate that the engineered enzyme has a drastically enhanced catalytic activity for non-methylated surrogates, confirming our computational data and resolved high-resolution X-ray structures at 1.55 Å resolution. Our combined findings provide crucial mechanistic understanding of the function of AsqJ and showcase how combination of computational and experimental data enables to rationally engineer enzymes.

## Abstract (web summary)

The catalytic activity of dioxygenase AsqJ is strictly relying on the methylation of quinolone substrates. Here, the authors apply molecular simulations, X-ray crystallography and in vitro biochemical studies to the engineering of dioxygenase AsqJ with improved catalytic activity for modified non-methylated surrogates.

## Introduction

**Fig. 1:**
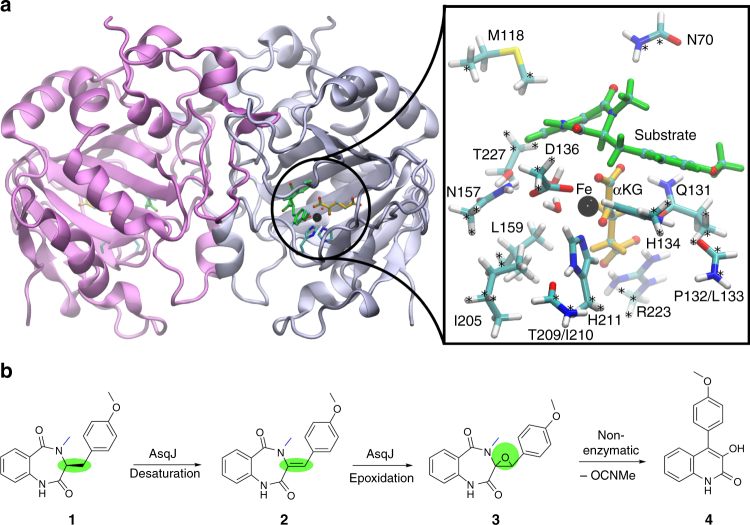
The structure and active site of AsqJ. **a** The dimeric protein structure of AsqJ (PDB ID:5DAQ; one subunit in the asymmetric unit), depicting the location of the active site. The inset shows a DFT model of the active site of AsqJ, comprising 194 atoms. Atoms marked with asterisk are kept fixed in the structure optimizations. **b** Reaction sequence catalyzed by AsqJ. Substrate **1** is the AsqK-produced NRPS product of anthranilic acid and Tyr(OMe); the N4-methyl group is derived from S-adenosylmethionine^15^

The non-heme Fe^II^/α-ketoglutarate-dependent dioxygenase AsqJ from *Aspergillus nidulans* (Fig. 1a) is an exceptional enzyme that activates dioxygen and stereoselectively catalyzes a C–C bond desaturation and epoxidation reaction^1,2^. AsqJ converts its natural substrate, 4′-methoxycyclopeptin (**1**), to a quinolone alkaloid, 4′-methoxyviridicatin (**4**) (Fig. 1b). It is remarkable that a single enzyme can catalyze such large-scale chemical transformations in a one-pot multistep reaction. The characteristic 4-arylquinolin-2(1*H*)-one structure of **4** is found in a variety of quinolone alkaloids^3–5^, which are compounds with promising antibacterial and antitumor activities^6^. In addition to the central role of non-heme Fe^II^/α-ketoglutarate-dependent oxygenases in many metabolic pathways^7–9^, elucidation of the catalytic mechanism of AsqJ is also important due to the promising application of different quinol alkaloids as potential drug candidates.

Watanabe and co-workers^1^ initially discovered AsqJ as the enzyme responsible for the biosynthesis of the quinolone alkaloid **4** in *Aspergillus nidulans* (Fig. 1). The crystal structure of the Ni^II^-substituted AsqJ was recently resolved at 1.7 Å resolution, revealing the molecular architecture of the dioxygenase in presence of different reaction intermediates^2^. The active site of AsqJ forms a funnel like reaction chamber, located at the interface between antiparallel β-strands. The substrate interacts with the active site metal, which is ligated by His-211, His-134, Asp-136, as well as by the C-2 keto group and C-1 carboxylate of α-ketoglutarate (αKG), and a crystallographic water molecule in an octahedral coordination sphere. Moreover, His-134 forms a π-stacking interaction with the substrate that may further play an important role for the substrate binding and the catalytic activity.

Although the catalytic cycle of AsqJ has remained unclear, it was suggested that the enzyme generates a highly oxidizing ferryl species by splitting dioxygen, similar to many other non-heme iron enzymes^10–12^. AsqJ inserts one of the oxygen atoms into αKG, yielding succinate upon decarboxylation^13^. The resulting ferryl is further employed to catalyze desaturation of the C–C single bond, yielding the intermediate 4′-methoxydehydrocyclopeptin (**2**), characterized in high-performance liquid chromatography–mass spectrometry (HPLC/MS) experiments^2^. Recent experimental and computational studies^14–16^ also support that the high-spin ferryl, Fe^IV^ = O, intermediate could indeed be the catalytically active species. In subsequent steps of the reaction cycle, binding of a second oxygen molecule is likely to induce epoxidation of the double bond of **2** leading to formation of the epoxide 4′-methoxycyclopenin (**3**), which in turn undergoes a non-enzymatic re-arrangement and elimination reaction to form the final product **4** (Fig. 1b). Interestingly, it was also observed that AsqJ can effectively catalyze the desaturation reaction only when the substrate is methylated at the N4 position (see Supplementary Fig. 1 for substrate labeling), whereas upon removal of the N4-methyl group, the epoxide **3** was not formed^2^.

From an evolutionary perspective, this finding is in line with the N4-methylation activity of the non-ribosomal peptide synthetase (NRPS) AsqK producing **1**^17^. Nevertheless, from a chemical point of view this finding is unexpected, since the methyl group is located three bonds apart from the reacting atoms in the substrate.

In order to probe the catalytic mechanics of AsqJ and to rationally engineer an enzyme that can catalyze chemical transformations of non-methylated substrates, we employ here an integrated computational and experimental approach. We derive the energetics and molecular structures of putative catalytic intermediates from multi-scale quantum and classical molecular simulations, which can provide powerful methodologies to study structure, dynamics, and energetics of complex (bio)chemical reactions on a wide range of timescales and spatial resolutions^18–21^. The computational work is combined with site-directed mutagenesis experiments, in vitro activity measurements by HPLC/MS, and structural characterization by X-ray crystallography.

## Results

### Energetics and mechanism of AsqJ

**Fig. 2:**
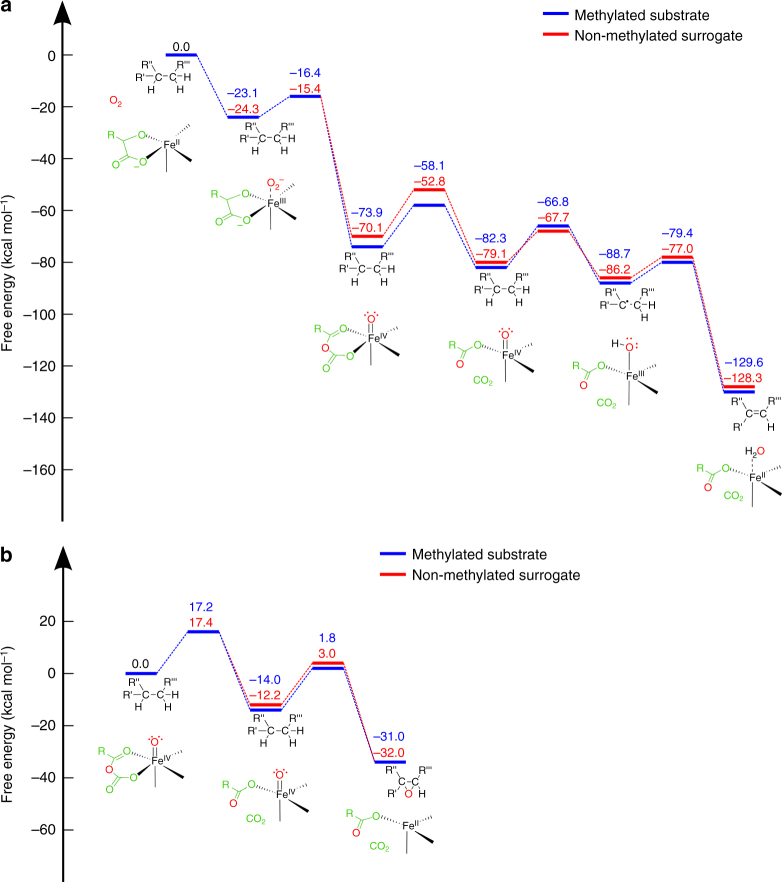
Calculated catalytic cycle and energetics of AsqJ. **a** The first part of the reaction cycle, comprising oxygen activation, decarboxylation of αKG, and two subsequent PCET reaction steps. **b** The second part of the reaction involving epoxidation of the substrate. All energies refer to free energies calculated in the quintet state at B3LYP-D3/def2-TZVP/ε = 4 level of theory with vibrational and entropic corrections at the B3LYP-D3/def2-SV(P)/ε = 4 level. Barriers are obtained from reaction pathway optimizations (Methods). See also Supplementary Movies 1 and 2, and Supplementary Figs. 3 and 9 for the optimized intermediate structures. Free energy profiles along the triplet surface are shown in Supplementary Figs. 6 and 7

In order to probe the energetics and structure of the reaction catalyzed by AsqJ for its natural substrate **1**, we performed quantum chemical density functional theory (DFT) calculations on active site enzyme models of AsqJ (Fig. 1a, inset). The catalytic cycle of AsqJ is initiated by binding of dioxygen to the Fe^II^ active site. In order to accommodate O_2_, a crystallographic water molecule observed in the Ni^II^-substituted structure was removed and the structures were re-optimized. The optimized structural model suggests that substitution of Ni^II^ with Fe^II^ results only in minor structural changes (Supplementary Fig. 2). The DFT free energies along the quintet pathway indicate that dioxygen binding is exergonic by ca. 23 kcal mol^−1^. Thus, formation of the ferric-superoxide species, Fe^III^–O_2_^•/−^, occurs spontaneously, similar as in many other iron enzymes (Fig. 2a, see Supplementary Figs. 6 and 7 for the triplet spin state)^22,23^. For the insertion of the oxygen atom into αKG, we obtain a barrier of ca. 7 kcal mol^−1^ with a transition state that has a peroxo-like character (Fig. 2a). For the subsequent decarboxylation step, a barrier of ca. 16 kcal mol^−1^ has to be overcome, for which the products CO_2_ and succinate are stabilized by hydrogen bonds with a crystallographic water molecule and Gln-131, respectively (Supplementary Fig. 3, 4). Moreover, the DFT calculations suggest that entropic effects do not considerably alter the reaction profiles, except for the oxygen binding and decarboxylation steps, for which we obtain *T*Δ*S* contributions at *T* = 298 K of ca. 6 kcal mol^−1^ (destabilizing) and 4 kcal mol^−1^ (stabilizing), respectively (Fig. 2a, Supplementary Fig. 5a).

The calculations suggest that the CO_2_ release could be coupled to a flip of the ferryl group towards the substrate, which also enables the reorientation of the CO_2_-stabilizing water molecule (Supplementary Movie 1). Notably, the achieved state is ca. 8 kcal mol^−1^ more favorable than the oxygen-inserted αKG state, and its configuration leads to an increase in the π-stacking interaction between the substrate and His-134, decreasing the distances between the groups from 3.7 Å to 3.5 Å. In the subsequent step, the oxidizing power of the ferryl is employed to thermodynamically drive two proton-coupled electron transfer (PCET) reactions, for which we obtain reaction barriers of ca. 16 kcal mol^−1^ and 9 kcal mol^−1^, for the first and second transfer reactions, respectively (Fig. 2a). The first PCET process results in a radical on C3 that delocalizes mainly on the C3–C2 bond (Supplementary Fig. 8a), whereas no significant spin density is observed on the proton during the transfer from C3 to the iron ligand. We therefore conclude that the proton and electron move along different reaction channels between the substrate and the redox-active metal (Supplementary Fig. 8b).

After the desaturation has taken place, the second part of the reaction cycle is initiated by binding of new αKG and O_2_ molecules, which lead to a similar decarboxylation and rotation of the oxo-ferryl bond, with a barrier of ca. 17 kcal mol^−1^ (Fig. 2b). We further find that the resulting ferryl species could attack the double bond with a barrier of 16 kcal mol^−1^, resulting in the subsequent formation of the epoxide and Fe^II^ (Fig. 2b, Supplementary Movie 2).

Taken together, the overall free energy profile is strongly exergonic, and the putative reactions have kinetically feasible barriers of ca. 7–17 kcal mol^−1^, which is consistent with the accumulation of the product on the seconds timescales in HPLC/MS experiments (see below). These barriers are also consistent with recent computational studies^15,16^, suggesting that the hydrogen abstraction and epoxidation reactions have barriers between 4 and 22 kcal mol^−1^.

### Substrate methylation is central for catalysis

**Fig. 3:**
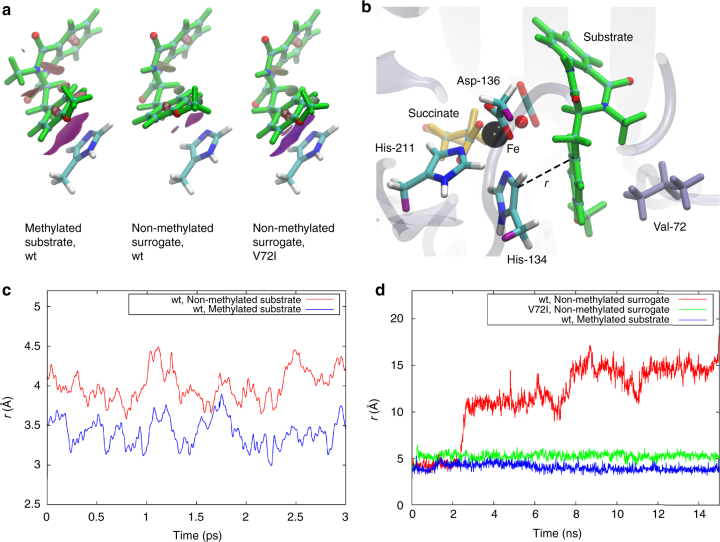
Engineering AsqJ to catalyze turnover of modified surrogates. **a** Non-covalent interaction densities (purple surfaces) between His-134 and the methylated substrate/non-methylated surrogate for wt and V72I-mutant AsqJ prior to the first PCET reaction. **b** Snapshot from a QM/MM MD simulation of AsqJ. The QM region is shown in colored licorice and link atoms are depicted in purple, and Val-72 is shown in gray licorice. **c** The stacking distance (*r*) between His-134 (CD2) and the substrate (C1′) in presence of the methylated substrate and the non-methylated surrogate after the first PCET reaction. **d** The stacking distance (*r*) in presence of the methylated substrate and the non-methylated surrogate for wt and V72I-mutant AsqJ before the first PCET reaction. The simulations were performed using both QM/MM MD simulations (**c**) and classical atomistic MD simulations (**d**) to explore different timescales

Next, we aimed to probe why AsqJ does not catalyze desaturation of the substrate analog lacking a methyl group at the N4 position. To this end we recalculated the reaction pathway with the non-methylated surrogate (Fig. 2, Supplementary Fig. 5, 9). Interestingly, we obtain similar energetics as for the natural substrate for most steps of the catalytic cycle. However, our computed binding affinities between the substrate and the protein, as well as the molecular strain energy stored within the substrates, suggest that there are significant differences between the N4-methylated and non-methylated educts (Table 1). We find that for the two PCET reactions, the methylated substrate undergoes marginal conformational changes around its C3–C1″–C1′–C2′ dihedral angle relative to its optimized structure in gas phase (Table 1, Supplementary Fig. 10). In stark contrast, for the non-methylated surrogate, we observe that the central C3–C1″–C1′–C2′ dihedral angle is twisted in the active site pocket by ca. 10–30° from its structure in gas phase (Table 1, Supplementary Fig. 10). According to the DFT calculations, these structural rearrangements introduces strain energy up to ca. 8 kcal mol^−1^ in the surrogate, which in turn weakens the π–π stacking interaction between the surrogate’s methoxy-phenyl group and His-134 (Table 1). This prediction is further supported by calculations of non-covalent interaction surfaces within the active site pocket (Fig. 3a), and it is also consistent with reduction in the binding affinities between the substrate and the active site during the PCET reactions (Table 1). These findings thus suggest that the stabilization of non-methylated surrogates in AsqJ is impaired during the PCET reactions.

**Table 1:** Binding affinities, strain energies, and dihedral angles (C3–C1″–C1′–C2′) for the substrates before and after the first and second PCET steps

	Methylated substrate before first PCET	Non-methylated surrogate before first PCET	Methylated substrate after first PCET	Non-methylated surrogate after first PCET	Methylated substrate after second PCET	Non-methylated surrogate after second PCET
Binding affinity [kcal mol^−1^]	T: −38.9 Q: −37.7	T: −32.7 Q: −38.0	T: −31.4 Q: −33.1	T: −30.9 Q: −32.6	T: −35.9 Q: −33.5	T: −31.2 Q: −30.5
Strain energy [kcal mol^−1^]	T: 0.2 Q: 0.2	T: 7.6 Q: 2.4	T: 0.1 Q: 0.1	T: 0.8 Q: 0.8	T: 0.0 Q: 0.0	T: 5.9 Q: 0.5
Dihedral angle gas-phase [deg]	−22	−72	−42	−71	−22	−20
Dihedral angle protein [deg]	T: −27 Q: −27	T: −39 Q: −52	T: −46 Q: −46	T: −59 Q: −59	T: −19 Q: −22	T: −52 Q: −11

T and Q refer to structures optimized in the triplet and quintet states, respectively.

We got interested in how this π-stacking interaction is influenced by the dynamics of the enzyme, and therefore performed hybrid quantum mechanics/classical mechanics (QM/MM) molecular dynamics (MD) simulations of the states with the native substrate and non-methylated surrogate after the initial PCET process. Consistent with the results from the DFT models, our computations reveal that the distance between the His-134 and the methoxy-phenyl ring of the substrate drastically increases from 3.4 to 4.0 Å from the native substrate to the non-methylated surrogate in the QM/MM MD simulations (Fig. 3b, c). Hence, our simulations support that the molecular strain within the non-methylated substrate might lead to its dissociation from the binding pocket during the PCET steps.

### Engineering AsqJ to catalyze non-methylated surrogate

In the QM/MM MD trajectories, we observe that Val-72, located on a loop surrounding the substrate-binding pocket, forms dispersive interactions with the methoxy-phenyl of the ligand and His-134 (Fig. 3b). In order to strengthen the π–π interactions between the non-methylated surrogate and the protein, we replaced the Val-72 with a somewhat larger isoleucine residue, followed by new DFT calculations and classical MD simulations. Interestingly, the DFT models suggest that the V72I replacement indeed strengthens the interaction between the protein and the methoxy-phenyl ring of the non-methylated substrate analog prior to the first PCET reaction by decreasing the distances between the aromatic systems from 4.1 to 3.8 Å (Fig. 3a). The MD simulations also indicate an enhanced stabilization of the non-methylated surrogate in the active site of V72I mutant (Fig. 3c). For comparison, we also studied the effect of the V72K, V72L, and F139I substitutions to increase the interaction between His-134 and the substrate from the opposite side. However, whereas the V72K and V72L mutants did not improve the π-stacking interactions (Supplementary Fig. 12), the F139I variant showed some minor increase in the dispersive interactions between His-134 and the surrogate.

**Fig. 4:**
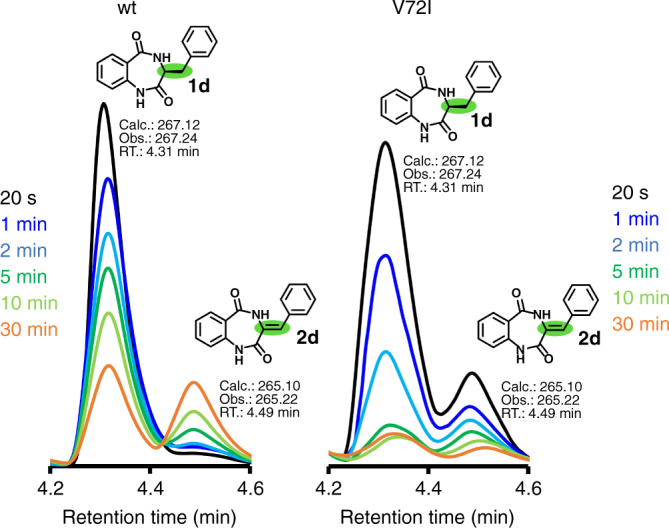
Activity measurements of wt and engineered AsqJ. Turnover of the wt and V72I-mutated AsqJ for the non-methylated substrate analog (**1d**) from reverse-phase HPLC/MS-coupled activity assays in the presence of Fe^II^, αKG, oxygen, and ascorbic acid. The absorbance at *λ *= 280 nm is shown for **1d** and the desaturated reaction intermediate **2d** (see Supplementary Fig. 13 for further characterization). The reaction progress was analyzed after 20 s (black trace), 1 min (dark blue), 2 min (light blue), 5 min (dark green), 10 min (light green), and 30 min (orange), showing that **2d** forms already after 20 s in V72I

In our next approach, we probed the engineered enzyme experimentally. To this end, we cloned, expressed, and purified the V72I-, V72K-, and F139I-mutated AsqJ enzymes. The catalytic activities of these mutants were determined for the native substrate **1**, cyclopeptine **1b **(Supplementary Fig. 13b), as well as for demethylcyclopeptine **1d** (Fig. 4) and compared to the wild-type (wt) AsqJ by using a reverse-phase HPLC/MS-coupled activity assay, and X-ray crystallography. The wt and V72I-, V72K-, F139I-mutated AsqJ variants turned out to have similar turnover rates for the methylated substrates **1** and **1b** (Supplementary Fig. 13a, b), suggesting that these alterations do not lead to a significant decrease in the enzyme activity. In the following, we analyzed how the engineered enzymes affect the turnover of the non-methylated substrate analog **1d**. Remarkably, the HPLC/MS activity assays depict that the desaturated reaction intermediate, **2d**, which follows the two PCET reactions of **1d** (Figs. 1b, 2a), is already formed at 20 s for V72I, in stark contrast to the wt AsqJ for which we observe the product peak with maximum intensity after 30 min (Fig. 4). In contrast, the F139I mutant only displays a minor increase in activity, whereas the turnover of V72K for **1d** is similar to the wt enzyme as proposed by our MD simulations (Supplementary Fig. 14). Notably, formation of the epoxide **3d** was not observed for the designed mutant V72I, implying that the mutated enzyme is capable of activating C–H bonds of **1d**, while it cannot effectively proceed into the second part of the reaction cycle. On the other hand, starting with the synthesized desaturated intermediate **2** of the methylated natural substrate **1**, the activity assays show similar turnover for the wt AsqJ and the V72I variant (Supplementary Fig. 13c, d). These results led us to conclude that the substrate methylation is also a fundamental prerequisite for the epoxidation reaction. These findings are supported by the QM/MM MD simulations, which indicate that stabilization of the non-methylated reaction intermediate **2d** inside the V72I variant is rather weak (Supplementary Fig. 15). Therefore, the following epoxidation, which further requires binding and activation of a new oxygen molecule and release of CO_2_, is prevented since the residence time of **2d** at the active center is probably far too short to successfully complete the oxidoreduction. However, elucidating the molecular principles for these steps and engineering an enzyme that would further catalyze the epoxidation reaction is outside the scope of the present work.

**Fig. 5:**
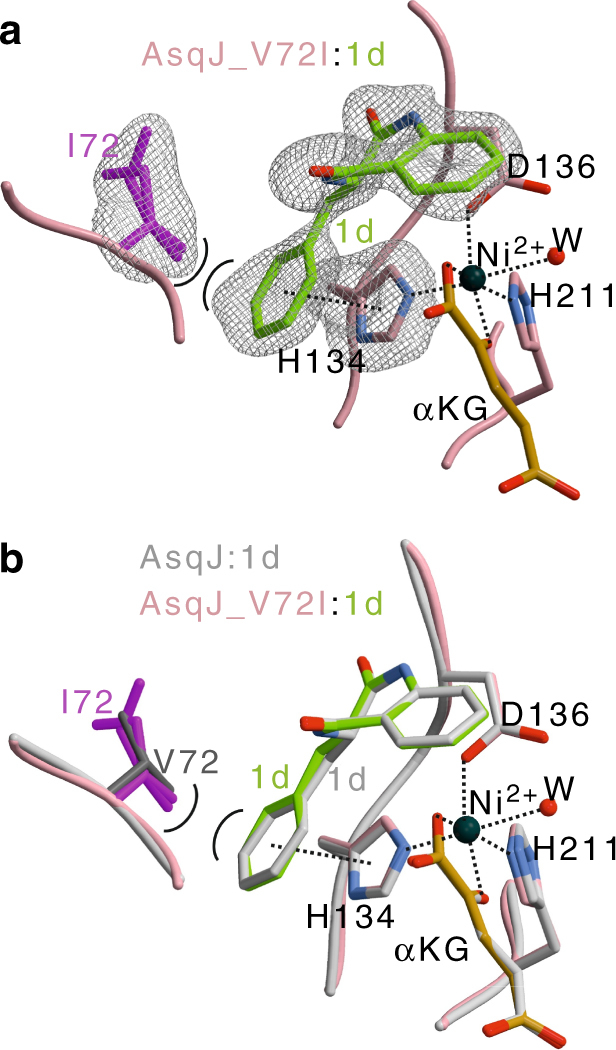
Crystal structure of the AsqJ_V72I mutant. **a**) Close-up of the active site in complex with **1d** (shown in green). The amino acids engaged in ligand binding are depicted as sticks and labeled by the one letter code, α-ketoglutarate (αKG) shown in orange, and Ni^II^ is shown as a black sphere. The electron density depicts a 2*F*_o_–*F*_c_ map with I72, H134, and **1d** omitted for phasing. I72 contains two alternative conformations with an occupancy of ~50% (PDB ID: 5OA8). An extra lobe of electron density around the phenyl ring of **1d** indicates increased mobility of the phenyl ring. **b** Structure of the active site superposition of AsqJ_V72I:**1d** with the wt AsqJ:**1d** (shown in gray; PDB ID: 5DAX). π-stacking and coordination of the metal atom are indicated by black-dotted lines. Note that the surrogate **1d** adopts identical positions in both crystal structures, while there are only marginal shifts in the distances of I72 coordinating the aromatic moiety of the ligand

Experimental structural insight into the function of AsqJ_V72I was obtained by crystallographically analyzing the mutant in complex with **1** (1.55 Å resolution, *R*_Free_ = 16.8%), **1b** (1.65 Å, *R*_Free_ = 17.4%), and **1d** (1.75 Å, *R*_Free_ = 19.3%), as well as determining the structure of V72K bound to **1b** (1.55 Å resolution, *R*_free_ = 19.4%, PDB ID 6EOZ). Our calculations predict that stabilization of these ligands in the active site depends on pronounced π-stacking interactions, which are most important for the initiation of the PCET reactions. Interestingly, the crystal structure of V72I in presence of **1d** reveals that the distance between Ile-72 and C4′ of the non-methylated analog is decreased by 0.2 Å compared to wt AsqJ:**1d** complex (PDB ID 5DAX, Fig. 5). Notably, despite the local conformational changes at the active site, all other parts of the mutant structure remain unchanged with a root-mean square deviation (RMSD) of 0.15 Å for the protein backbone atoms. These experimental insights perfectly match to our initial computational refinements (see above). Moreover, a close inspection of the electron density map depicts an alternative conformation rotation of the Ile-72-side chain, positioning the introduced isobutyl group in an inward and outward orientation (Fig. 4). In addition, the B-factor of **1d** is increased from 28.7 (AsqJ-wt) to 35.3 Å^2^ (AsqJ-V72I) (Supplementary Table 3, Supplementary Fig. 16). This observation accounts for an improved mobility of the phenyl group of **1d** around the C3–C1″ bond in the AsqJ_V72I, and is caused by the two alternative conformations of Ile-72. Consistently, an extra lobe of electron density around the phenyl ring of **1d** in the AsqJ_V72I structure indicates that the aromatic moiety of the ligand is quite flexible (Fig. 5a). Thus, our findings suggest that the stable π-stacking interactions of **1d** with His-134 lead to a prolonged residence time of the ligand at the active site, facilitating the desaturation reaction. Taken together, our findings suggest that substrate binding in AsqJ is linked with a conformational rearrangement of the residues in the loop surrounding the specificity pocket, and stabilization of the different conformational states might provide a promising starting point for future studies to rationally engineer AsqJ to catalyze the turnover of modified substrates.

In conclusion, the molecular mechanism of an exceptional one-pot multistep dioxygenase AsqJ from *A. nidulans* was studied by combined molecular simulations, biochemical in vitro activity assays, mutagenesis, and X-ray crystallography. The enzyme catalyzes a stepwise desaturation and epoxidation reaction of 4′-methoxycyclopeptin to the quinolone alkaloid, 4′-methoxyviridicatin by highly exergonic and kinetically feasible proton-coupled transfer PCET reactions. We observed that methylation of the substrate at the N4 position is important for stabilizing dispersive interactions within the active site during the PCET processes. Combining various orthogonal methodologies such as multi-scale computational chemistry, mutagenesis, in vitro activity assays, and protein crystallography, revealed that an engineered AsqJ_V72I mutant is able to rapidly catalyze desaturation of the non-methylated surrogate, which is in stark contrast to wt AsqJ. We could show that the smallest possible V72I replacement in a loop surrounding the active site drastically improved turnover of demethylcyclopeptin **1d**, and that insertion of one methyl group to the protein compensates for the lack of one *N*-methyl group in the substrate. In conclusion, the achieved results provide an important starting point to understand molecular mechanisms of multifunctional enzymes, and to rationally engineer such systems for the synthesis of natural products with important biomedical applications.

## Methods

### DFT models

Quantum chemical DFT cluster models with 190–194 atoms were constructed based on our recently crystallized Ni^II^-substituted model of AsqJ (PDB ID: 5DAQ)^2^. The QM models comprised in addition to the iron-oxo species the substrate, αKG (or CO_2_ + succinate), two crystal water molecules, and amino acid residues Asn-70, Met-118, Gln-131, His-134, Asp-136, Asn-157, Leu-159, Ile-205, His-211, Arg-223, and Thr-227 (Fig. 1a, inset). We note that, inclusion of Val-72 in the QM models did not considerably change the relative energies between the reaction intermediates (Supplementary Table 1). The amino acids were cut between the Cα and Cβ atoms, except for Asn-70, Thr-227, and Arg-223, which were cut between Cβ/Cγ and Cγ/Cδ atoms, respectively. We also included the backbone of residues Pro-132/Leu-133 and Thr-209/Ile-210. The terminal carbon atoms were saturated with hydrogen atoms, and fixed during structure optimization to account for steric effects of the protein environment (Fig. 1a, inset). Geometry optimizations were performed at dispersion-corrected DFT level^24^, using the B3LYP-D3 functional^25,26^, and def2-SVP (light elements) and def2-TZVP basis sets (Fe). The protein surroundings were treated as a polarizable medium with a dielectric constant of *ε* = 4 using the COSMO model^27,28^. Reaction pathways were optimized using the Woelfling method^29^, a chain-of-states method that is related to the nudged-elastic band^30^ and zero-temperature string methods^31^. The final energetics for all states were computed at the B3LYP-D3/def2-TZVP/ε = 4 level, and electronic configurations were analyzed from Mulliken populations and spin density distributions. The reaction pathways were optimized for both the triplet and quintet spin states, as also supported by recent computational studies suggesting that several spin states may contribute in the catalysis steps of AsqJ^15,16^. The absolute electronic energies for the quintet states were ca. 5–10 kcal mol^−1^ more stable than for the triplet states, whereas the structural and relative energy differences between the states were marginal. Vibrational and entropic corrections were obtained at the B3LYP-D3/def2-SV(P)/ε = 4 level by estimating the molecular Hessian numerically^32^. Furthermore, we studied the isolated substrate in different states by both complete and restrained optimizations at the B3LYP-D3/def2-SVP level. Strain energies were obtained from restrained optimizations of the relaxed substrates to their protein geometries. All distances and free energies discussed in the main text and shown in the figures were extracted from the optimized QM models. All QM calculations were performed using TURBOMOLE v. 6.6^33^, and non-covalent interaction regions were calculated using NCIPLOT^34^.

### Combined quantum mechanical and molecular mechanical methods

Hybrid QM/MM MD simulations of AsqJ with the substrate with and without the methyl group at the N4 position, prior and after the first PCET reaction, were performed at the B3LYP-D3/CHARMM36^35^ level using def2-SVP (light elements) and def2-TZVP (Fe) basis sets. The QM region comprised protein residues His-134, Asp-136 and His-211, the succinate/CO_2_ moiety, in addition to the ferryl (Fe^IV^ = O^2-^) or ferric (Fe^III^–OH^−^) metal center and the substrate. Link atoms were introduced between the Cβ and Cα atoms of the protein residues, and the monomeric protein model was embedded in a water box with 18,089 water molecules, and neutralized by 100 mM NaCl. The complete QM/MM system, comprising 58,896 atoms, was simulated for 3 ps for each state at *T* = 310 K, and by using a 1 fs integration time step. No positional restrains were applied in the QM/MM MD simulations. All QM/MM calculations were performed using TURBOMOLE v. 6.6 linked together with CHARMM^36,37^.

### Classical MD simulations

Classical MD simulations of the wt and V72I, V72K, and F139I in silico mutants were performed for 15 ns at *T* = 310 K using a 1 fs time step, and treating the long-range electrostatics using the Particle Mesh Ewald (PME) approach^38^. We employed the CHARMM36 force field, together with force field parameters for the active site models based on CGenFF^39^ and by calculated restrained electrostatic potential (RESP) charges for the substrates and small active site models at the B3LYP/def2-TZVP level obtained with NWChem^40^. Classical MD simulations were performed using NAMD^41^, and visual molecular dynamics was used for visualization analysis^42^.

### Site-directed mutagenesis experiments and protein purification

Mutations in the wt *asqJ* gene of the fungus *A. nidulans* FGSC A4 (pET28bSUMO*An*AsqJ, GenBank: XP_682496.1)^2^ were introduced via the QuikChange Site-Directed Mutagenesis Kit (Agilent Technologies) using oligonucleotides listed in Supplementary Table 2. Bacterial culture and protein purification were performed according to published procedures^2^. The protein solutions were buffer exchanged to 20 mM Tris hydrochloride, pH 7.4, containing 100 mM NaCl and 2 mM dithiothreitol, and were concentrated to a concentration of 20 mg/mL using a 10k Amicon Ultra Centrifugal Filter Device (Millipore, Billerica, MA), flash frozen with liquid nitrogen and stored at −80 °C.

### HPLC/MS experiments

Reaction mixtures containing 33 µM purified AsqJ_wt (AsqJ_V72I, AsqJ_V72K, or, AsqJ_F139I), 200 µM of the synthesized substrate^2^, 100 µM FeSO_4_, 2.5 mM α-ketoglutarate, 4 mM ascorbic acid, and 50 mM Tris hydrochloride were incubated at 30 °C for 20 s, 1 min, 2 min, 5 min, 10 min, 30 min, and 60 min. The reaction was stopped by adding 10% (v/v) of 3 M trichloroacetic acid, and samples were centrifuged at 10,000 ×  *g* for 10 min. All traces were monitored at 280 nm using a Dionex UltiMate 3000 HPLC system coupled with a Thermo LCQ fleet in combination with a Waters 1525 binary HPLC pump, X-Bridge Prep C18 column (5 µm, 10 × 250 mm), Waters 2998 PDA detector and Waters Fraction Collector III (Waters).

### X-ray crystallography

Mutant AsqJ proteins were crystallized as previously described for the wt AsqJ^2^. Crystals were grown at 20 °C using the sitting drop vapor diffusion method. Drops contained a 1:1 mixture of protein solution (20 mg ml^−1^ protein, 1 mM α-ketoglutarate as well 2 mM **1**, **1b**, or **1d**) and reservoir solution (100 mM Tris hydrochloride, pH 7.8–8.2, 1.0 M LiBr, 27–30% PEG6000). Crystals were cryoprotected by addition of cryobuffer (100 mM Tris hydrochloride, pH 7.8, 500 mM LiBr, 30% PEG400). Diffraction data were collected at the beamline X06SA at the Paul Scherrer Institute, SLS, Villigen, Switzerland (*λ* = 1.0Å). Evaluation of reflection intensities and data reduction were performed with the program package XDS^43^. Positioning of the initial model was carried out with the coordinates of the AsqJ_wt (PDB ID: 5DAQ) by rigid body refinements (REFMAC5^44^). MAIN^45^ and COOT^46^ were used to build models. TLS (Translation/Libration/Screw) refinements finally yielded excellent *R*_work_ and *R*_free_, as well as RMSD bond and angle values (Supplementary Table 3). Note, the asymmetric unit of the crystals contains one subunit, which by crystallographic symmetry forms the physiological dimer, with two identical active sites.

### Data availability

The coordinates, proven to have good stereochemistry from the Ramachandran plots, were deposited at the RCSB Protein Data Bank under the accession codes 5OA4 (AsqJ_V72I:**1**), 5OA7 (AsqJ_V72I:**1b**), 5OA8 (AsqJ_V72I:**1d**), and 6EOZ (AsqJ_V72K:**1b**). All other data is available from the authors upon reasonable request.

## Electronic supplementary material

Supplementary Information(PDF 1504 kb)

Description of Additional Supplementary Files(PDF 165 kb)

Supplementary Movie 1(GIF 5879 kb)

Supplementary Movie 2(GIF 2780 kb)
