# Supplementary material for: Catalytic mechanism and molecular engineering of quinolone biosynthesis in dioxygenase AsqJ
Source: Nat Commun. 2018 Mar 21;9:1168. doi: 10.1038/s41467-018-03442-2 (PMC5862883; doi:10.1038/s41467-018-03442-2)
Supplement: Supplementary file 1 — Supplementary Information(PDF 1504 kb) [file 41467_2018_3442_MOESM1_ESM.pdf]

Supplementary Information

Catalytic Mechanism and Molecular Engineering of Quinolone Biosynthesis in Dioxygenase AsqJ

Mader et al.

Supplementary Figures

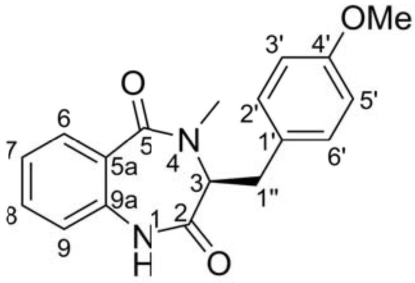

(*S*)-3-(4'-methoxybenzyl)-4-methyl-3,4-dihydro-1*H*-benzo[1,4]diazepine-2,5-dione

Supplementary Figure 1. Substrate numbering.

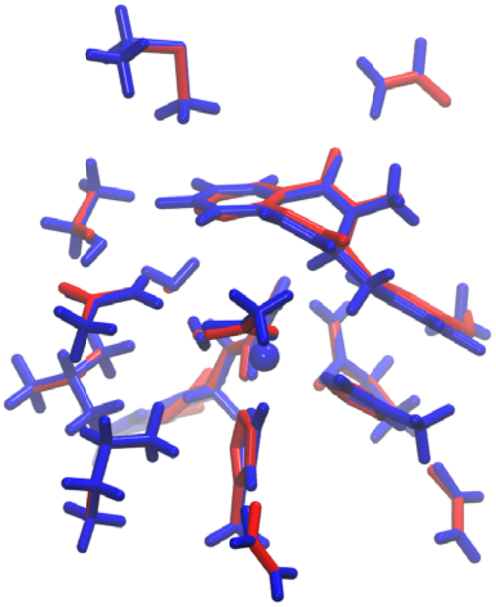

Supplementary Figure 2. Optimized structural models of AsqJ with Fe^{II} (in blue) compared to the crystal structure with Ni^{II} (in red).

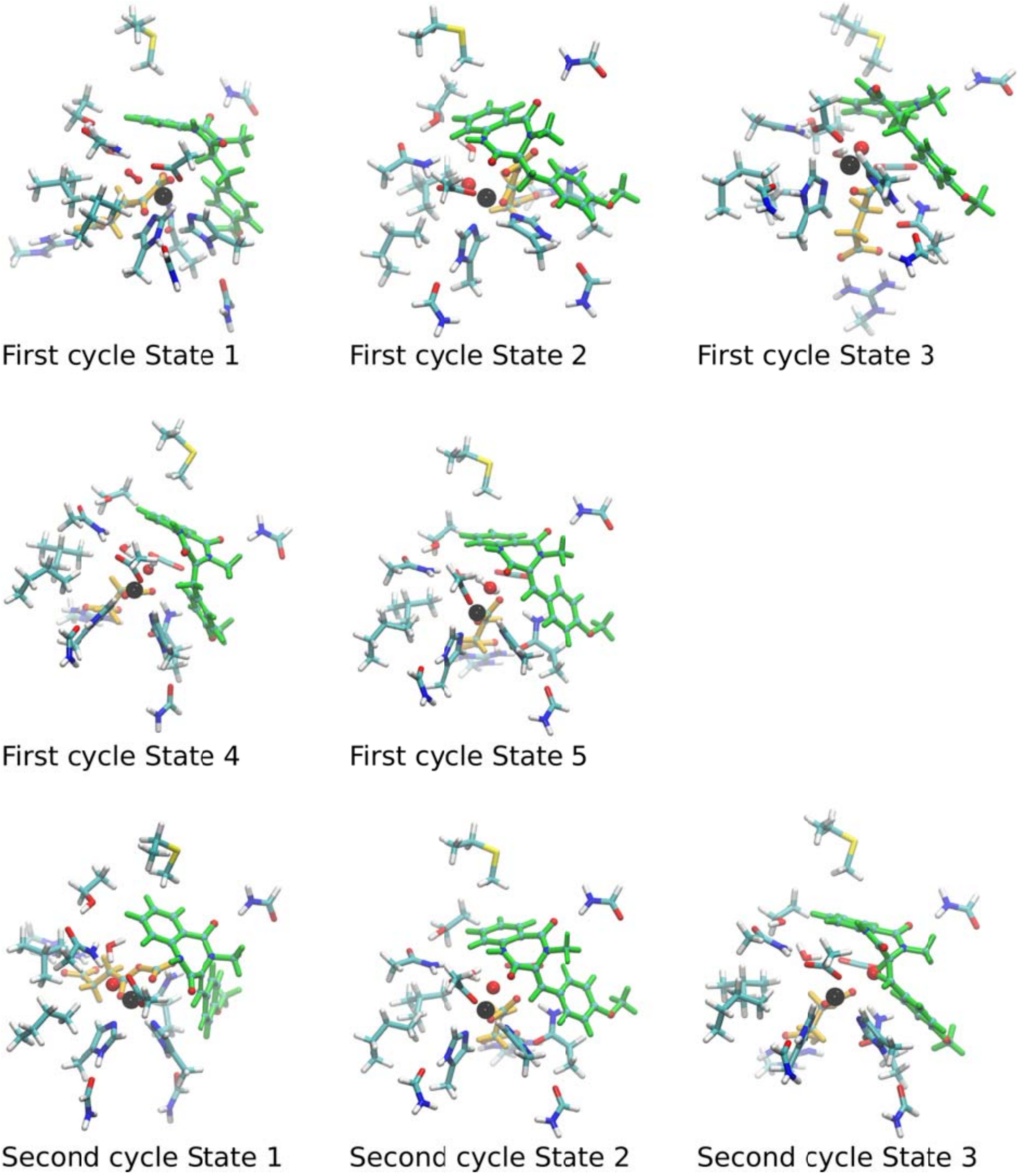

Supplementary Figure 3. DFT optimized structures of putative catalytic cycle intermediates for the methylated substrate (**1**) shown in green. Starting with dioxygen bound to the iron (state 1), the dioxygen molecule is split and one of the oxygen atoms is inserted into αKG (shown in yellow) (state 2), yielding succinate upon decarboxylation (state 3). The resulting ferryl abstracts two hydrogen atoms from the substrate, leading to a radical (state 4), followed by the desaturated intermediate (state 5). Upon binding of new αKG and dioxygen molecules, the dioxygen molecule is split in the same way as in steps 1→2 of the first reaction cycle (second cycle, state 1), leading to succinate after decarboxylation (second cycle, state 2). The ferryl species finally adds its oxygen to the double bond, forming an epoxide (second cycle, state 3). The shown models were optimized in the triplet state, and they are structurally very similar to the quintet state structures (see below for optimized coordinates).

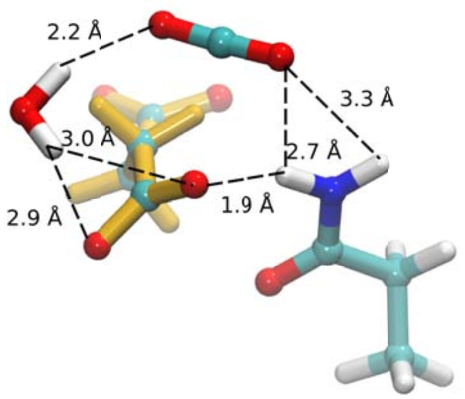

Supplementary Figure 4. Distances between decarboxylation products, CO_2 and succinate, a crystallographic water molecule, and Gl**n**-131.

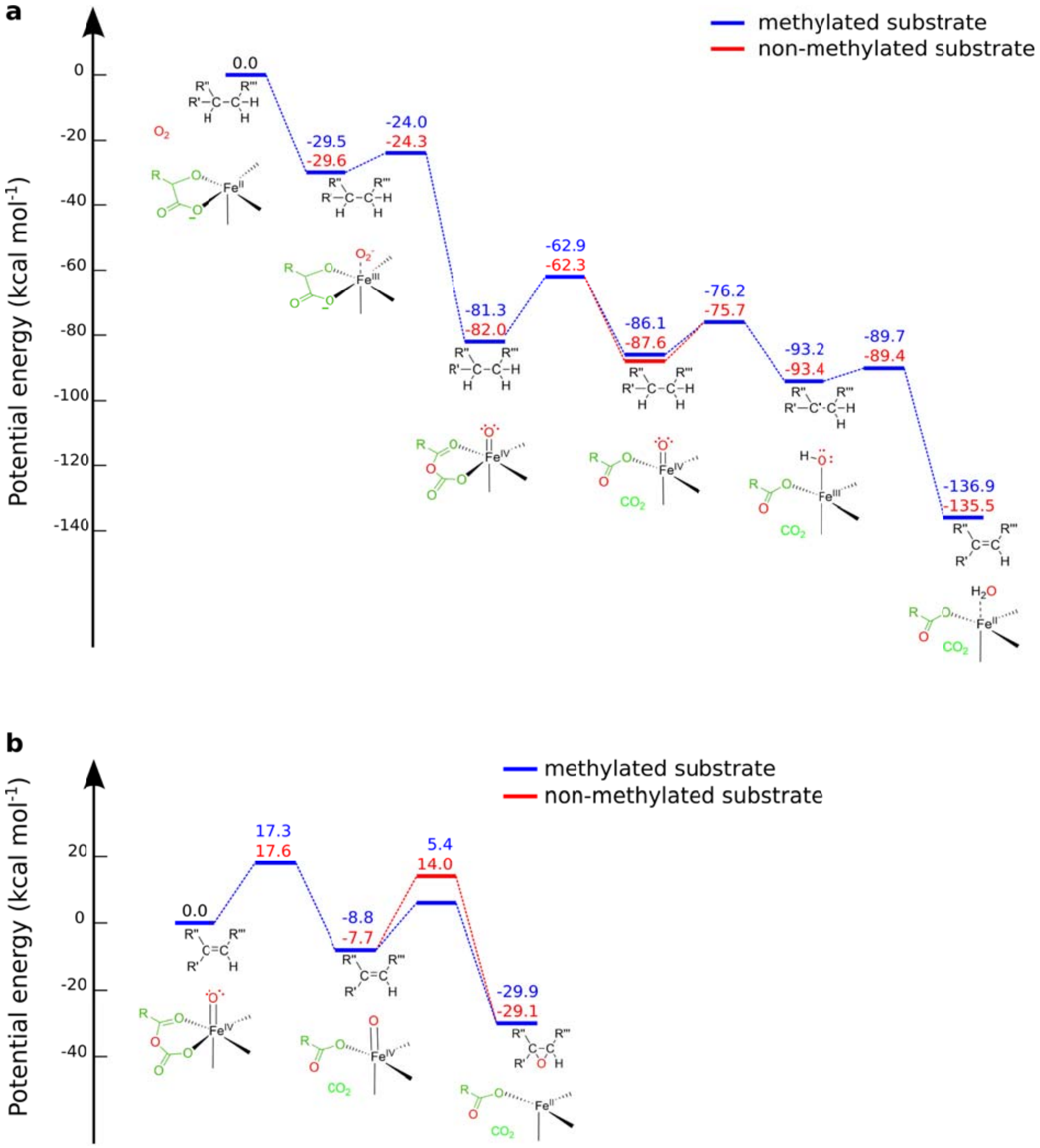

Supplementary Figure 5. Electronic energy profiles in the quintet state for the putative catalytic cycle of AsqJ, comprising **a)** substrate desaturation and **b)** substrate epoxidation. Corresponding free energy profiles are shown in the main text Fig. 2.

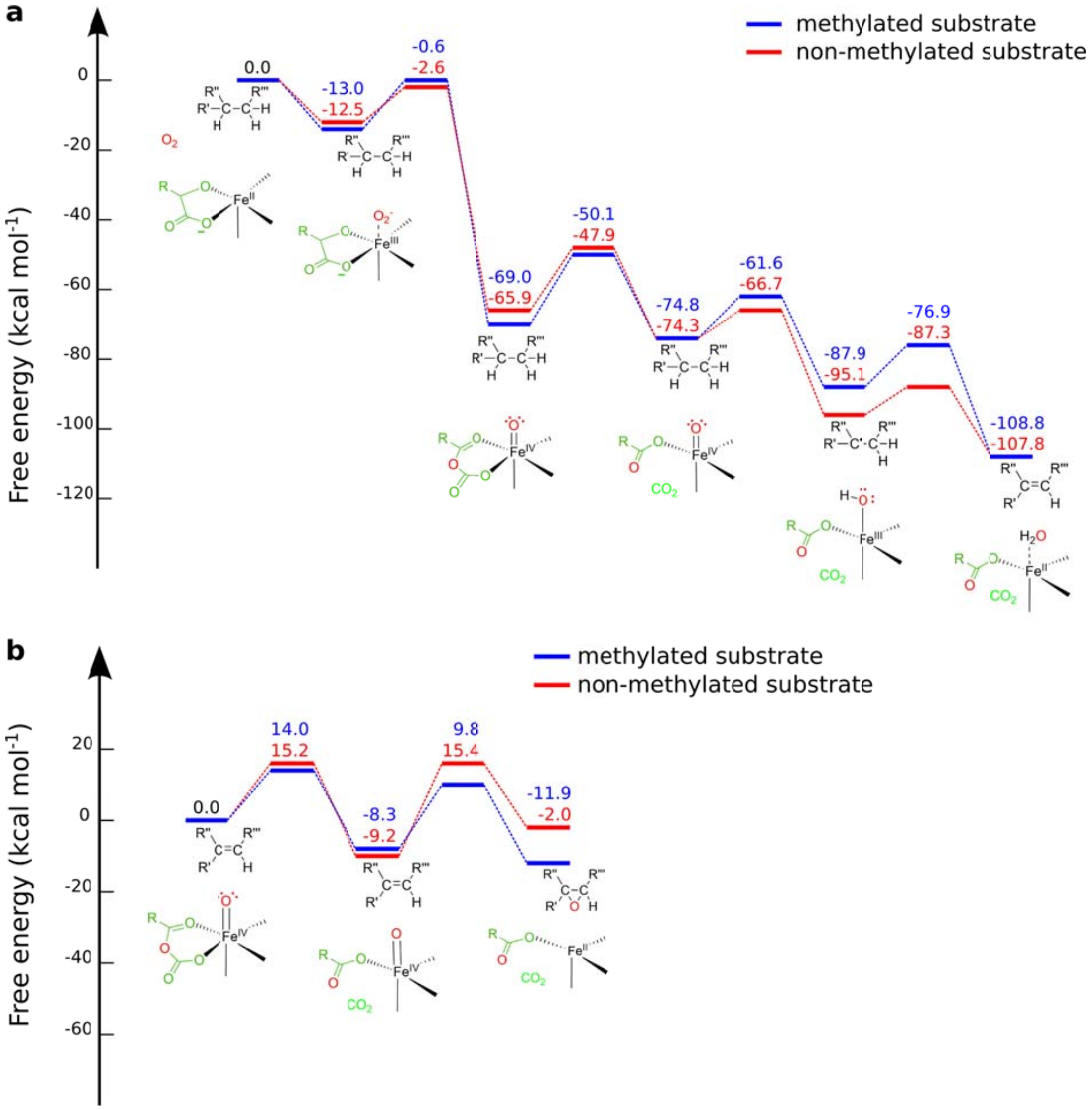

Supplementary Figure 6. Free energy profiles in the triplet state for the putative catalytic cycle of AsqJ, comprising **a)** substrate desaturation and **b)** substrate epoxidation.

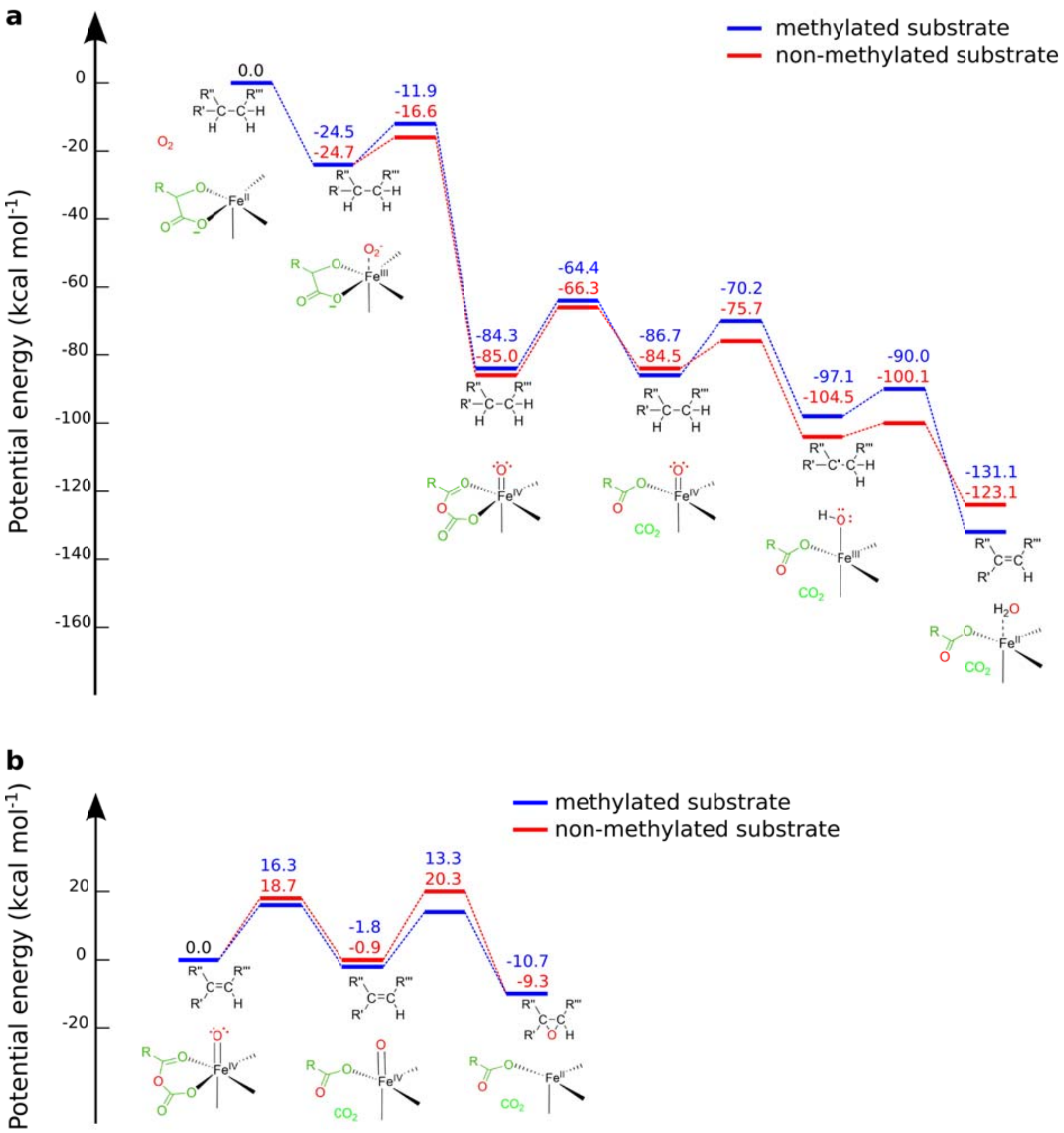

Supplementary Figure 7. Electronic energy profiles in the triplet state for the putative catalytic cycle of AsqJ, comprising **a)** substrate desaturation and **b)** substrate epoxidation. Corresponding free energy profiles are shown in the Supplementary Fig. 6.

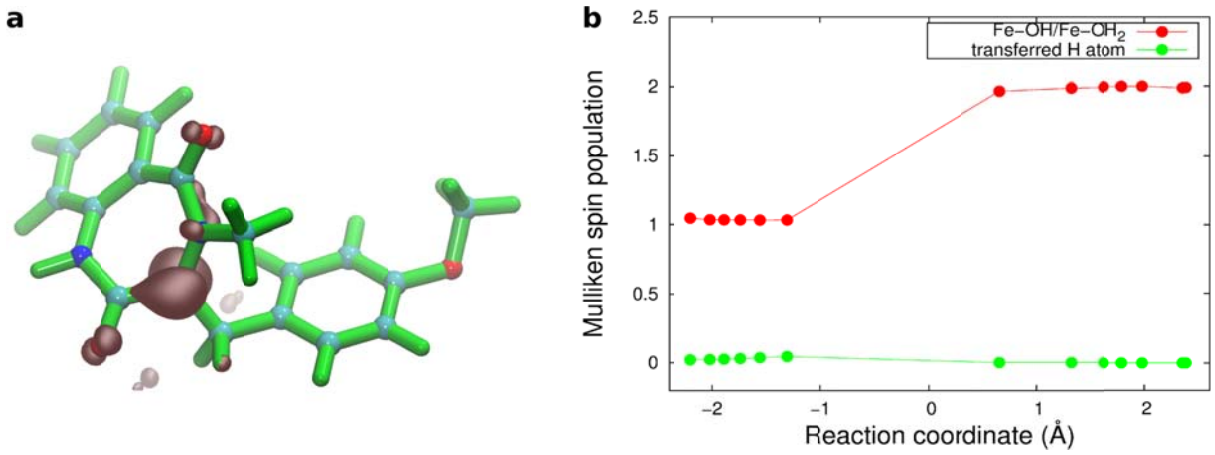

Supplementary Figure 8. **a)** Spin distribution (in brown) on the substrate (in green) after the first PCET process. **b)** Mulliken spin populations as a function of the *r*(C-H) - *r*(OH-Fe) reaction coordinate during the second PCET process on the Fe-OH/Fe-OH_2 moiety in the triplet state (in red) and on the transferred hydrogen atom (in green).

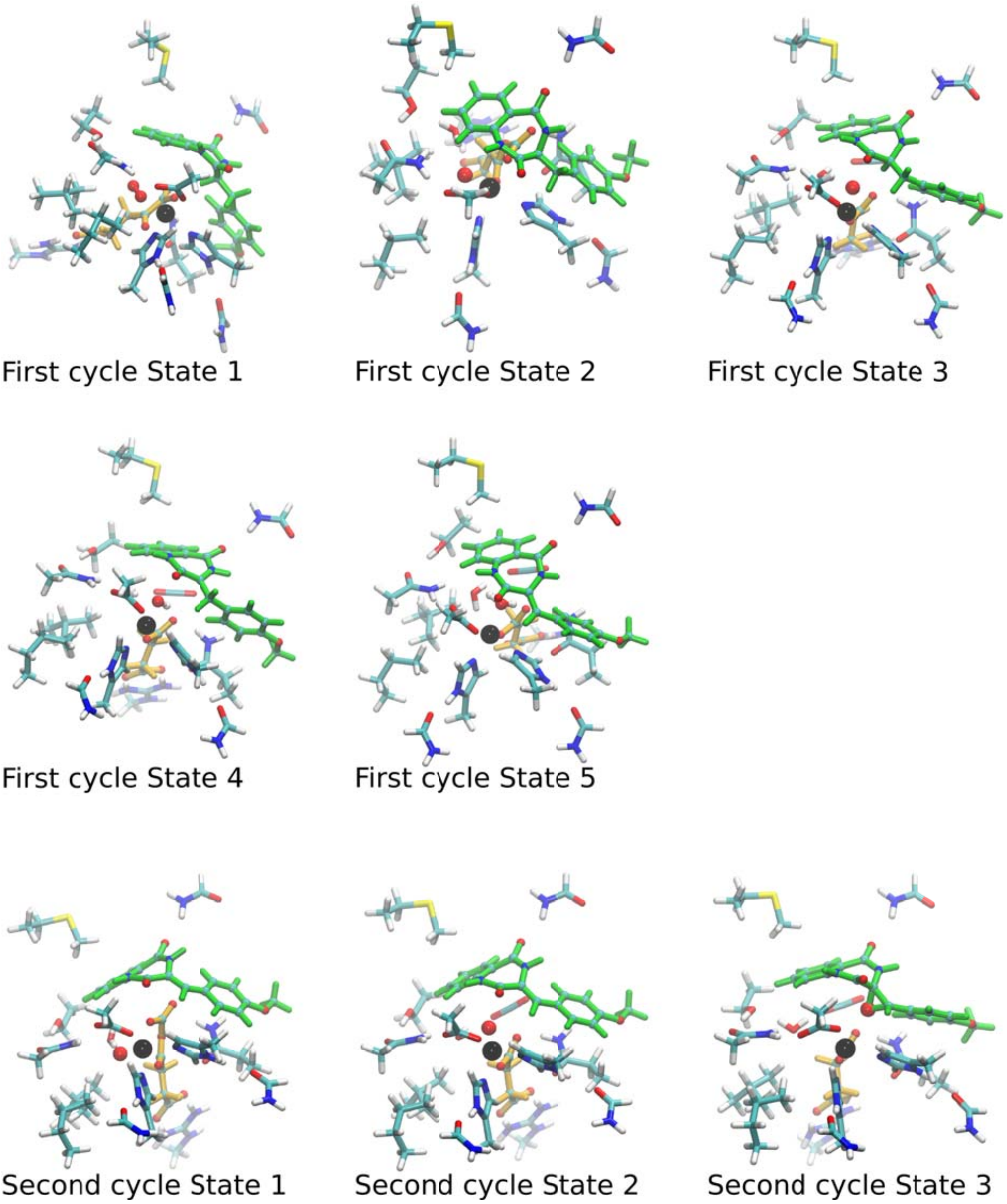

Supplementary Figure 9. DFT optimized structures of putative catalytic cycle intermediates for the methylated substrate (**1d**) shown in green. Starting with dioxygen bound to the iron (state 1), the dioxygen molecule is split and one of the oxygen atoms is inserted into αKG (shown in yellow) (state 2), yielding succinate upon decarboxylation (state 3). The resulting ferryl abstracts two hydrogen atoms from the substrate, leading to a radical (state 4), followed by the desaturated intermediate (state 5). Upon binding of new αKG and dioxygen molecules, the dioxygen molecule is split in the same way as in steps 1→2 of the first reaction cycle (second cycle, state 1), leading to succinate after decarboxylation (second cycle, state 2). The ferryl species finally adds its oxygen to the double bond, forming an epoxide (second cycle,

state 3). The structures shown were optimized in the triplet state (see attached coordinates for structures optimized in the quintet state).

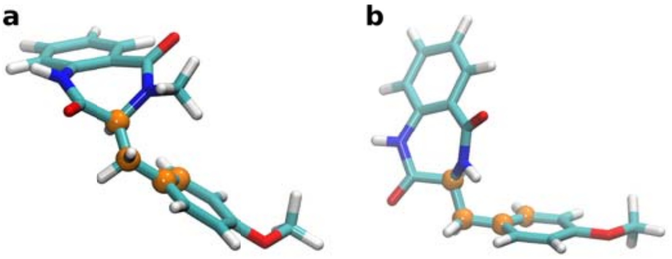

Supplementary Figure 10. Gas-phase optimized structures of **a**) methylated substrates and **b**) non-methylated surrogate with the C3-C1''-C1'-C2' dihedral angle indicated in orange.

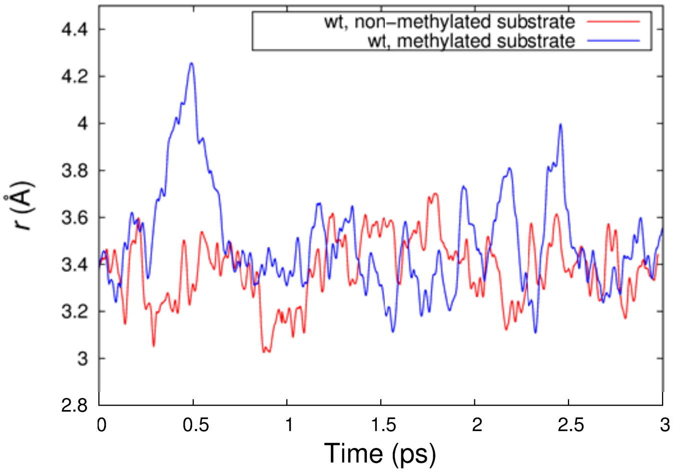

Supplementary Figure 11. QM/MM MD simulations of AsqJ with methylated substrates and non-methylated surrogate before the first PCET.

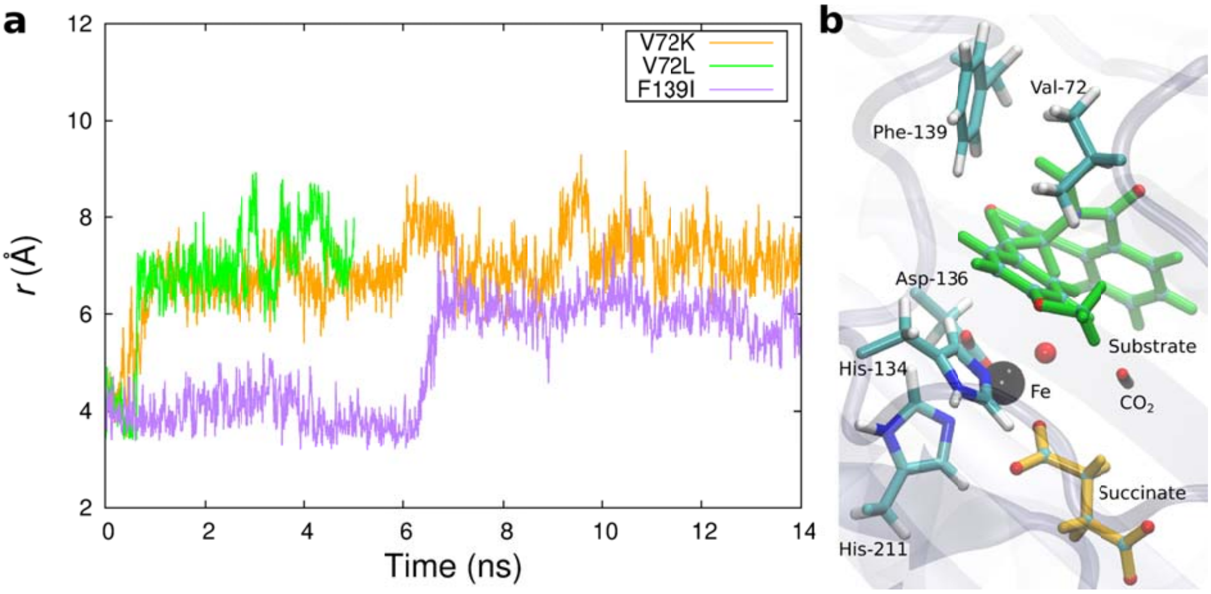

Supplementary Figure 12. **a**) Classical MD simulations of AsqJ-V72K, AsqJ-V72L, and AsqJ-F139I showing π-stacking distances between the non-methylated surrogate and His-134 before the first PCET. **b**) Active site of AsqJ, showing the location of Val-72 and Phe-139.

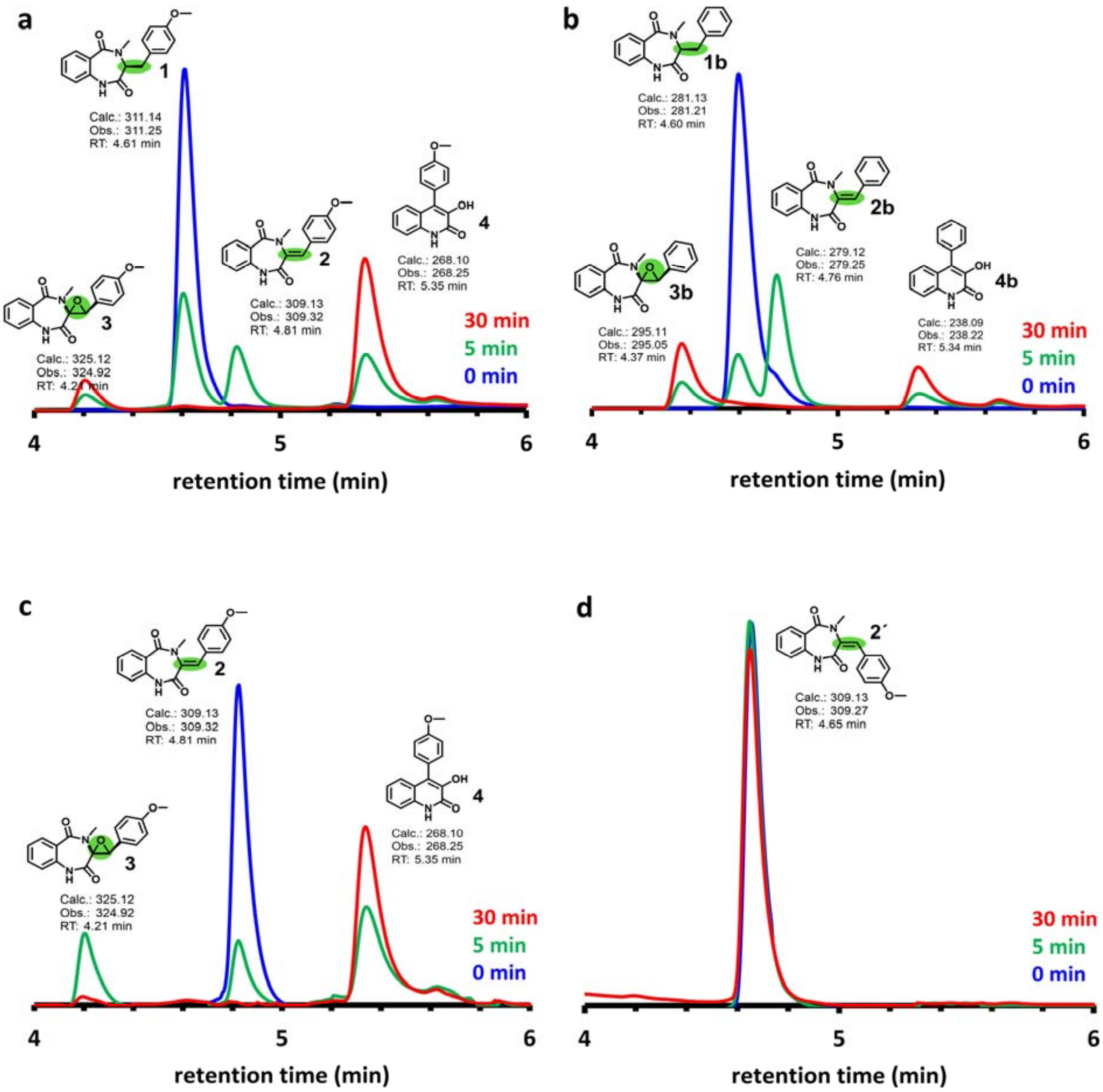

Supplementary Figure 13. Data from reverse phase HPLC/MS coupled activity assay for the AsqJ-V72I mutant in the presence of Fe^{II}, αKG, oxygen, and ascorbic acid showing λ=280 nm absorption for **a)** substrate **1**, two reaction intermediates **2** and **3**, and final product **4**. The reaction progress was analyzed after 0 min (blue graph), 5 min (green), and 30 min (red). **b)** Using the substrate analog **1b** that lacks the methoxy group also revealed the two reaction intermediates **2b** and **3b**, as well as the product **4b**. Thus, the methoxy group is not essential for catalysis. **c, d**) Starting with the synthesized intermediate **2** (Z-isomer) or **2`** (E-isomer) reveals that only the Z-isomer is transformed into **3** and **4**. Turnover is similar for AsqJ_wt, AsqJ_V72I, AsqJ_V72K, AsqJ_F139I; results of AsqJ_V72I are shown.

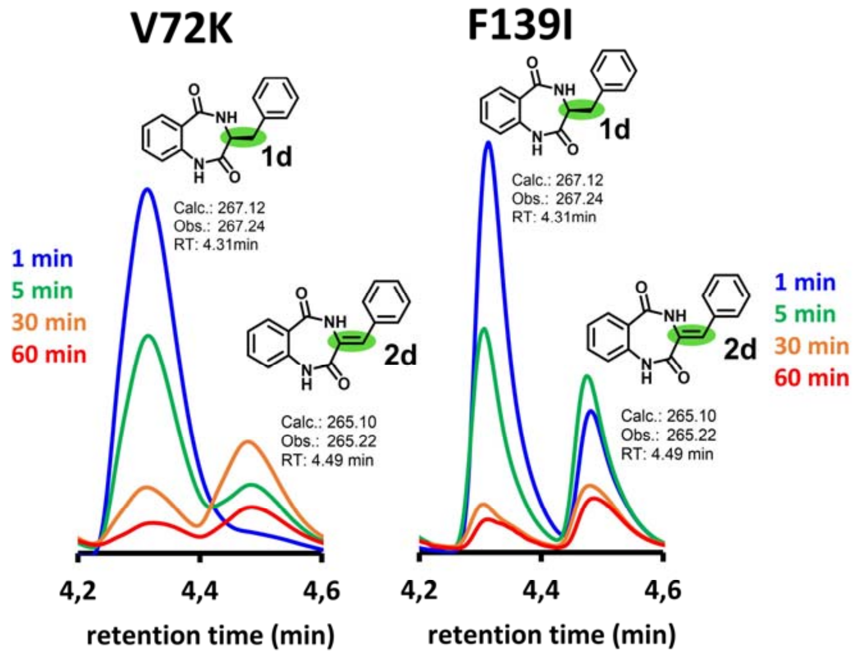

Supplementary Figure 14. Data from reverse phase HPLC/MS coupled activity assay for V72K and F139I mutants of AsqJ in the presence of Fe^{II}, αKG, oxygen, and ascorbic acid showing λ=280 nm absorption for the non-methylated substrate analog **1d** and the desaturated reaction intermediate **2d.** The reaction progress was analyzed after 1 min (blue graph), 5 min (green), 30 min (orange), and 60 min (red). The F139I mutant also shows some increased activity in comparison to the wt (Fig. 4), whereas the turnover of V72K for **1d** is similar to the wt enzyme.

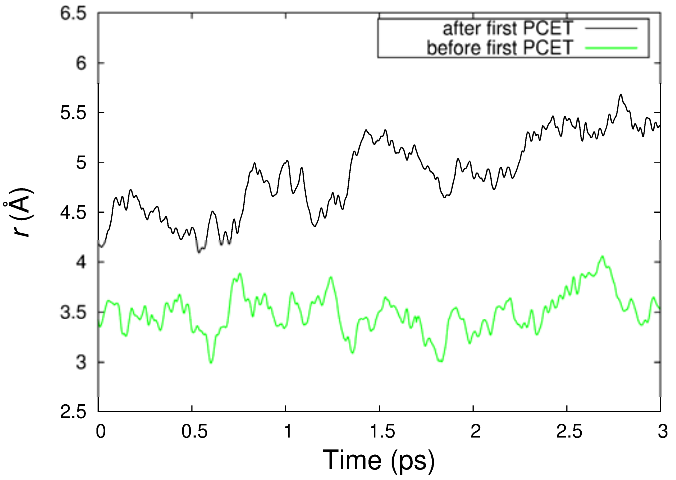

Supplementary Figure 15. QM/MM MD simulations of the AsqJ-V72I mutant with the non-methylated surrogate before and after the first PCET.

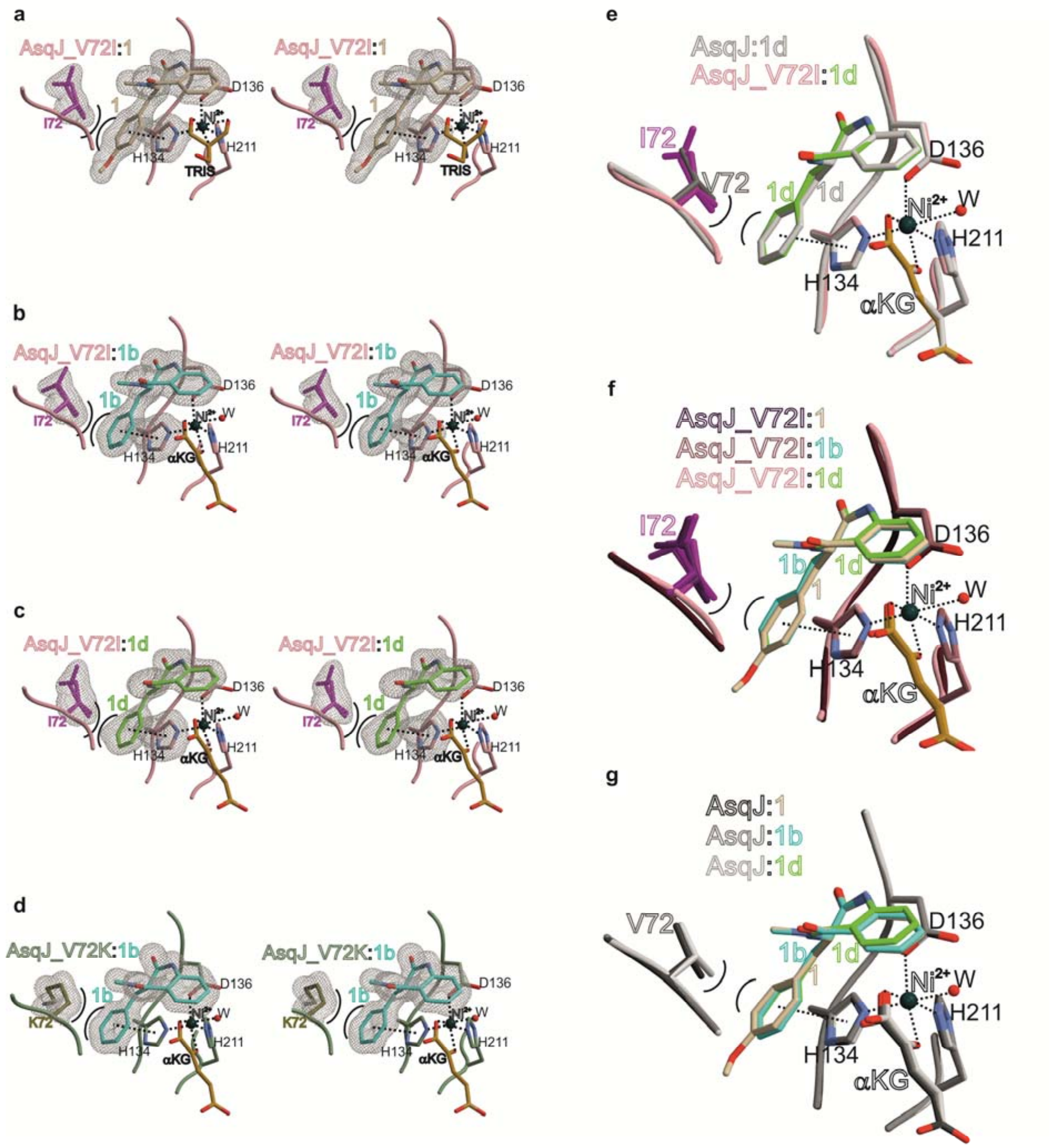

Supplementary Figure 16. Stereo representation of electron density maps and structural overlays of wild type and AsqJ mutants. Structure of **a)** AsqJ_V72I:**1** (PDB ID: 5OA4; notably, a Tris molecule coordinates the Ni^{2+} rather than the expected αKG), **b)** AsqJ_V72I:**1b** (PDB ID: 5OA7), **c)** AsqJ_V72I:**1d** (PDB ID: 5OA8) and **d)** AsqJ_V72K:**1b** (PDB ID: 6EOZ) complexes. The $2F_O$-F_C electron density map for I72 (alternative conformation) / K72, ligands, and H134 is contoured at 1σ (grey mesh). **e-g)** Structural superposition of **e)** AsqJ:**1d** (PDB ID: 5DAX)[1] and AsqJ_V72I:**1d**, **f)** V72I:**1**, V72I:**1b** and AsqJ_V72I:**1d** as well as AsqJ:**1** (PDB ID: 5DAQ)[1] AsqJ:**1b** (PDB ID: 5DAW)[1], and AsqJ:**1d**, respectively.

Supplementary Tables

Supplementary Table 1. Relative potential energies (in kcal mol^{-1}) for the QM cluster models of AsqJ with and without Val-72.

	Methylated substrate, without V72 (kcal mol^{-1})	Methylated substrate, with V72 (kcal mol^{-1})	Non-methylated substrate, without V72 (kcal mol^{-1})	Non-methylated substrate, with V72 (kcal mol^{-1})
**State 1**	0.0	0.0	0.0	0.0
**State 2**	-60.4	-60.2	-60.3	-60.2
**State 3**	-62.7	-62.4	-59.8	-62.0
**State 4**	-73.1	-72.6	-79.8	-79.2
**State 5**	-107.1	-106.3	-100.5	-102.6

Supplementary Table 2. Primer sequences used in this study.

**Oligonucleotide**	**Sequence 5` → 3`**
V72I_for	5`-ggtcgtggtcgataagatgtgattcggatggtctt-3`
V72I_rev	5`-aagaccatccgaatcac*atc*ttatcgaccacgacc-3`
V72K_for	5`-ggcgggtcgtggtcgataacttgtgattcggatggtctttc-3`
V72K_rev	5`-gaaagaccatccgaatcac*aag*ttatcgaccacgacccgcc-3`
F139I_for	5`-caatcggatgggagatgcgcatatcgcgatg-3`
F139I_rev	5`-catcgcgatatgcgc*atc*tcccatccgattg-3`

Supplementary Table 3. X-ray data collection and refinement statistics.

	AsqJ_V72I:1	*AsqJ_V72I:1b*	*AsqJ_V72I:1d*	*AsqJ_V72K:1b*	*AsqJ:1d*[1]
**Crystal parameters**					
Space group	$C222_1$	$C222_1$	$C222_1$	$C222_1$	$C222_1$
Cell constants	a = 72.8 Å<br>b = 120.9 Å<br>c = 66.8 Å	a = 72.8 Å<br>b = 119.9 Å<br>c = 67.1 Å	a = 73.1 Å<br>b = 120.1 Å<br>c = 67.0 Å	a = 72.6<br>b = 120.1<br>c = 67.2	a = 73.3 Å<br>b = 118.1 Å<br>c = 67.3 Å
Subunits / AU[a]	1	1	1	1	1
**Data collection**					
Beam line	X06SA, SLS	X06SA, SLS	X06SA, SLS	X06SA, SLS	X06SA, SLS
Wavelength (Å)	1.0	1.0	1.0	1.0	1.0
Resolution range (Å)[b]	50 – 1.55 (1.65 - 1.55)	50 – 1.65 (1.75 - 1.65)	50 – 1.75 (1.85 - 1.75)	50 – 1.55 (1.65 - 1.55)	50 – 1.7 (1.6 - 1.7)
No. observations	161411	164442	156353	208494	167592
No. unique reflections[c]	42587	35034	29922	42404	31808
Redundancy	3.8 (3.8)	4.7 (4.6)	5.2 (5.3)	4.9 (4.9)	5.3 (5.3)
Completeness (%)[b]	98.9 (99.5)	98.0 (99.5)	99.3 (99.4)	98.7 (99.7)	97.9 (98.3)
R_{merge} (%)[b, d]	4.0 (53.9)	4.3 (54.9)	5.4 (56.2)	4.2 (49.3)	4.1 (43.5)
R_{pim} (%)	4.6 (62.3)	4.8 (61.6)	6.1 (62.4)	4.7 (55.5)	4.5 (48.3)
CC1/2	1.0 (0.80)	1.0 (0.85)	1.0 (0.87)	1.0 (0.90)	1.0 (0.94)
I/σ (I)[b]	17.2 (2.4)	16.1 (2.3)	16.4 (2.6)	19.2 (3.4)	20.8 (3.4)
**Refinement (REFMAC5)**					
Resolution range (Å)	15 - 1.55	15 - 1.65	15 - 1.75	15 -1.55	15 - 1.7
No. refl. working set	40403	33229	28366	40230	30216
No. refl. test set	2127	1749	1492	2118	1591
No. non hydrogen	2606	2550	2464	2562	2523
No. substrate/surrogate	23	21	20	21	20
Solvent (H_2O, Ions, Tris)	344	285	195	301	265
R_{work}/R_{free} (%)[e]	13.9 / 16.8	13.8 / 17.4	15.7 / 19.3	13.7/16.3	18.1 / 19.4
r.m.s.d. bond (Å) / (°)[f]	0.006 / 1.3	0.007 / 1.3	0.007 / 1.3	0.007 / 1.4	0.005 / 1.2
Average B-factors ($Å^2$)					
Protein	25.9	34.9	33.9	23.4	31.1
Ligand	23.3	28.3	35.3	18.1	28.7
Solvent	27.5	45.0	39.8	34.2	37.9
Ramachandran Plot (%)[g]	97.6 / 2.4 / 0.0	97.6 / 2.4 / 0.0	97.6 / 2.4 / 0.0	97.6 / 2.4 / 0	97.6 / 2.4 / 0.0
PDB accession code	5OA4	5OA7	5OA8	6EOZ	5DAX

[a] Asymmetric unit

[b] Values in parentheses indicate the resolution range, completeness, R_{merge} and I/σ (I) correspond to the highest resolution shell

[c] Data reduction was carried out with XDS from a single crystal. Friedel pairs were treated as identical reflections

[d] $R_{merge}(I) = \Sigma_{hkl}\Sigma_j \mid I(hkl)_j - \langle I(hkl)\rangle \mid / \Sigma_{hkl} \Sigma_j I(hkl)_j$, where $I(hkl)_j$ is the jth measurement of the intensity of reflection hkl and <I(hkl)> is the average intensity

[e] $R = \Sigma_{hkl} \mid |F_{obs}| - |F_{calc}| \mid / \Sigma_{hkl} |F_{obs}|$, where R_{free} is calculated without a sigma cut off for a randomly chosen 5% of reflections, which were not used for structure refinement, and R_{work} is calculated for the remaining reflections

[f] Deviations from ideal bond lengths / angles

[g] Number of residues in favored / allowed / outlier region

Supplementary Table 4. Coordinates of the QM cluster model of state 1 of the first reaction sequence, optimized with quintet ferryl.

C	-27.2579843	-17.3879901	0.5119999
O	-27.2361474	-18.3221101	1.3014957
N	-26.7989845	-17.4109891	-0.7359998
C	-25.4809858	-15.4299917	-10.2089941
C	-24.9545421	-14.4803996	-9.1360328
S	-25.4048687	-14.9381218	-7.4171220
C	-24.4029851	-16.4449914	-7.2049960
C	-19.3309886	-25.5119848	1.3099996
C	-18.9694369	-24.0317915	1.1484525
C	-19.4329143	-23.4511454	-0.1877305
O	-19.4515325	-24.1347530	-1.2130349
N	-19.7963864	-22.1509216	-0.1889341
C	-20.7219882	-29.7009847	0.9539997
O	-21.1337110	-28.7923941	0.2404675
N	-20.4449883	-30.9339813	0.5429998
C	-23.7809863	-28.8239839	-2.5189983
C	-22.9411074	-27.6459021	-2.8915397
N	-21.9711457	-27.1062244	-2.0645996
C	-21.4212494	-26.0347521	-2.6795202
N	-21.9773588	-25.8606613	-3.8742008
C	-22.9232430	-26.8585593	-4.0218442
C	-24.6379861	-24.9089860	-8.3089957
C	-23.2343991	-24.6029812	-7.8007608
O	-23.0458565	-24.7022813	-6.5081542
O	-22.3493286	-24.3484792	-8.6151885
C	-20.5389882	-22.6719886	-11.8559937
C	-20.2880829	-21.7087475	-10.6898672
O	-19.6899933	-20.6340376	-10.8633340
N	-20.6684513	-22.1270261	-9.4696590
C	-15.0539918	-21.4899880	-9.5369943
C	-16.4567516	-21.9426950	-9.0926018
C	-16.5785318	-23.4700939	-9.0197325
C	-16.8376343	-21.2994096	-7.7514760
C	-18.1399889	-27.3639853	-11.9339926
C	-18.8208679	-26.1282744	-11.3167329
C	-19.2944375	-26.3184547	-9.8732150
C	-16.7943885	-27.7235646	-11.3022314
C	-20.8929881	-31.3979842	-9.0979954
O	-20.8442590	-30.2218597	-8.7528557
N	-20.5799882	-32.4509839	-8.3489957
C	-17.9129890	-28.8779849	-6.3399962
C	-19.0102384	-27.8781379	-6.4473831
N	-20.1910399	-28.1341518	-7.1158788
C	-20.9855144	-27.0552696	-7.0044085
N	-20.3845612	-26.1125462	-6.2891238
C	-19.1459264	-26.6088522	-5.9342719
C	-9.8599942	-19.9459874	-1.6139985
N	-11.1042640	-20.3456472	-0.9573314
C	-12.2333892	-20.7688716	-1.5814384
N	-13.3495518	-20.9647323	-0.8754292
N	-12.2572697	-21.0022210	-2.8938417
C	-18.9116140	-17.8087952	-8.5765842
O	-19.7041447	-18.9920201	-8.5874014
C	-19.5839885	-16.7449923	-7.7059958
Fe	-21.4041566	-24.4779016	-5.4509985
O	-14.5659016	-22.0723088	-3.9541831
C	-15.5891810	-22.1769496	-3.2249004
O	-15.6793087	-21.7746083	-2.0308553
C	-16.8192667	-22.9000735	-3.8164921
C	-18.1459985	-22.2022188	-3.5206150
C	-19.3772141	-22.9791073	-3.8748483
O	-19.3778152	-24.0905835	-4.3965655
C	-20.7716635	-22.3566193	-3.5860468
O	-21.7317621	-22.9645098	-4.1866337
O	-20.8851610	-21.4035499	-2.8177509
C	-22.3848210	-23.9683412	2.7216724
O	-22.9088375	-25.0411312	1.9606402
C	-23.3777144	-24.7742634	0.7055450
C	-24.3036662	-25.6861738	0.1744589
C	-24.8473253	-25.4663519	-1.0878810
C	-22.9898017	-23.6666578	-0.0586989
C	-23.5283616	-23.4728850	-1.3368726

C	-24.4796399	-24.3539787	-1.8674674
C	-25.1038141	-24.1859717	-3.2346504
C	-24.9410440	-22.8244540	-3.8978477
C	-25.5547684	-22.8320516	-5.2973961
O	-26.3738633	-23.6512678	-5.6885750
N	-25.1221484	-21.7999400	-6.0850628
C	-24.1634035	-20.8175265	-5.7572149
C	-23.2594160	-20.4346313	-6.7634174
C	-22.3058349	-19.4481019	-6.5317172
C	-22.2391457	-18.8282903	-5.2760337
C	-23.1325897	-19.1980330	-4.2763932
C	-24.1032706	-20.1928272	-4.4869417
C	-25.0899150	-20.4148453	-3.3707458
O	-25.4727964	-19.4571185	-2.6909633
N	-25.5326439	-21.6909925	-3.1614768
C	-26.6829579	-21.9265473	-2.2982505
O	-20.5296992	-23.1607447	-6.7045895
O	-19.1019812	-19.4452016	-5.7399927
H	-26.8598945	-16.5876521	-1.3240929
H	-26.3745291	-18.2486883	-1.1420466
H	-25.2051848	-15.0661371	-11.2137437
H	-26.5784919	-15.5115944	-10.1579303
H	-25.0588219	-16.4413948	-10.0962021
H	-25.3718782	-13.4690770	-9.2702105
H	-23.8563026	-14.3866363	-9.1924287
H	-24.5275686	-16.7821542	-6.1654267
H	-23.3339425	-16.2392001	-7.3759230
H	-24.7264484	-17.2590019	-7.8713606
H	-18.9550823	-25.9118855	2.2637569
H	-18.8661787	-26.0718787	0.4749929
H	-20.4255373	-25.6896618	1.2525246
H	-19.3550508	-23.4221254	1.9817181
H	-17.8705565	-23.9121865	1.1687891
H	-20.1226778	-21.7257752	-1.0634090
H	-19.7581715	-21.5864024	0.6502128
H	-20.5425284	-29.5514166	2.0447298
H	-20.5637549	-31.1812881	-0.4356764
H	-20.1060597	-31.6421429	1.1845467
H	-24.4660352	-29.0624743	-3.3457238
H	-24.4006471	-28.6411570	-1.6147705
H	-23.1680045	-29.7282327	-2.3185603
H	-20.6423706	-25.4120618	-2.2376309
H	-23.5310262	-26.9291498	-4.9209237
H	-24.8256788	-24.3064556	-9.2095831
H	-25.4503619	-24.7602890	-7.5706189
H	-24.6501171	-25.9724827	-8.6278366
H	-20.6883396	-22.1283397	-12.7958504
H	-21.3771820	-23.3507164	-11.6099607
H	-19.6042805	-23.2672985	-11.8929707
H	-21.1617175	-23.0016205	-9.2719995
H	-20.3817953	-21.5890503	-8.6537000
H	-14.7485859	-21.9309668	-10.5097980
H	-14.2929198	-21.7637054	-8.7850749
H	-15.0475888	-20.3853663	-9.6502913
H	-17.1826008	-21.5838127	-9.8477687
H	-15.8966732	-23.8824311	-8.2542069
H	-16.3270932	-23.9452220	-9.9837485
H	-17.6038899	-23.7736350	-8.7506350
H	-16.1251996	-21.5851425	-6.9560382
H	-17.8422094	-21.6062320	-7.4231400
H	-16.8359675	-20.1989966	-7.8174487
H	-18.8434677	-28.2219101	-11.8377377
H	-18.0165179	-27.1855564	-13.0248953
H	-19.6890190	-25.8581143	-11.9440218
H	-18.1296181	-25.2660515	-11.3689367
H	-19.9333447	-27.2135364	-9.7864722
H	-19.8817618	-25.4579631	-9.5148971
H	-18.4537272	-26.4494676	-9.1727280
H	-16.9021168	-27.9627768	-10.2310674
H	-16.0835754	-26.8827541	-11.3807612
H	-16.3361294	-28.5980824	-11.7930543
H	-21.2240072	-31.6753661	-10.1244115
H	-20.2672135	-32.3261838	-7.3897208
H	-20.6494195	-33.3938935	-8.7156773
H	-18.2537938	-29.7949967	-5.8284870
H	-17.0753052	-28.4559340	-5.7534394
H	-17.5093621	-29.1856698	-7.3292359

H	-21.9768253	-26.9813387	-7.4403755
H	-18.4584515	-26.0249282	-5.3301025
H	-10.0595842	-19.1386476	-2.3501383
H	-9.1634654	-19.5752640	-0.8527024
H	-9.3891324	-20.8044142	-2.1394754
H	-11.1845100	-20.1567978	0.0345498
H	-14.2557501	-21.2686787	-1.3430190
H	-13.3647671	-20.7322776	0.1099259
H	-11.4123360	-20.9309161	-3.4453078
H	-18.7566162	-17.4335908	-9.6059314
H	-17.9076811	-18.0335521	-8.1636871
H	-19.5680637	-19.5080594	-9.4166336
H	-20.3909363	-16.2010895	-8.2370617
H	-18.8533613	-16.0052610	-7.3431340
H	-20.0371974	-17.2640752	-6.8364101
H	-13.1385956	-21.3914190	-3.3350918
H	-16.6784584	-23.0344275	-4.8986685
H	-16.8448578	-23.9060240	-3.3610990
H	-18.2067717	-21.9329159	-2.4542561
H	-18.2270588	-21.2479475	-4.0746917
H	-21.6965662	-27.4920535	-1.1562575
H	-22.2018872	-24.3567748	3.7332617
H	-23.0995371	-23.1273199	2.7749106
H	-21.4329152	-23.5929702	2.3136373
H	-24.5932860	-26.5489998	0.7787862
H	-25.5821088	-26.1749300	-1.4820785
H	-22.2538406	-22.9552145	0.3138404
H	-23.1786870	-22.6154802	-1.9145029
H	-24.6380915	-24.9005129	-3.9315998
H	-26.1663966	-24.4703882	-3.2051824
H	-23.8634437	-22.6317174	-4.0040261
H	-25.4445723	-21.8325968	-7.0490231
H	-23.2952835	-20.9443974	-7.7300727
H	-21.5996747	-19.1823288	-7.3218670
H	-21.4763623	-18.0734359	-5.0776364
H	-23.1012990	-18.7173413	-3.2971967
H	-27.4684653	-22.4661169	-2.8516170
H	-27.0748771	-20.9586237	-1.9668270
H	-26.4029743	-22.5193790	-1.4140732
H	-19.7845844	-19.9976565	-5.3348715
O	-20.0311796	-22.0371584	-6.3484611
H	-27.6869399	-16.3918218	0.7905063
H	-19.2458842	-19.6039632	-6.6922620
H	-20.4143430	-28.9877946	-7.6512815

Supplementary Table 5. Coordinates of the QM cluster model of state 2 of the first reaction sequence, optimized with quintet ferryl.

C	-27.2579763	-17.3879861	0.5119999
O	-27.3191699	-18.3448358	1.2708402
N	-26.7989775	-17.4109851	-0.7359998
C	-25.4809788	-15.4299877	-10.2089911
C	-24.9358422	-14.4822713	-9.1437482
S	-25.3841754	-14.9247731	-7.4204178
C	-24.4029781	-16.4449874	-7.2049940
C	-19.3309826	-25.5119768	1.3099996
C	-19.0274470	-24.0161986	1.1536697
C	-19.6152961	-23.4594400	-0.1421245
O	-19.5094167	-24.0795112	-1.2006933
N	-20.2439377	-22.2659965	-0.0610355
C	-20.7219832	-29.7009767	0.9539997
O	-21.0816004	-28.7789995	0.2299934
N	-20.4449823	-30.9339723	0.5429998
C	-23.7809803	-28.8239759	-2.5189973
C	-22.9588138	-27.6378069	-2.9300077
N	-21.9768649	-27.0771610	-2.1311134
C	-21.4378926	-26.0166465	-2.7759811
N	-22.0143430	-25.8651201	-3.9649436
C	-22.9626688	-26.8663203	-4.0766943
C	-24.6379781	-24.9089780	-8.3089927
C	-23.2314707	-24.6231956	-7.7835640
O	-23.0528263	-24.8181058	-6.5045239
O	-22.3573969	-24.3168531	-8.5939407
C	-20.5389822	-22.6719826	-11.8559897
C	-20.2961622	-21.7295407	-10.6736395

O	-19.7398766	-20.6303952	-10.8307570
N	-20.6336235	-22.1947423	-9.4561603
C	-15.0539878	-21.4899820	-9.5369913
C	-16.4397940	-21.9451029	-9.0542208
C	-16.5927753	-23.4708020	-9.0596366
C	-16.7208458	-21.3709836	-7.6595726
C	-18.1399849	-27.3639773	-11.9339886
C	-18.8232964	-26.1321291	-11.3119222
C	-19.2834083	-26.3261791	-9.8641480
C	-16.7932896	-27.7236369	-11.3044118
C	-20.8929821	-31.3979752	-9.0979934
O	-20.8611935	-30.2238309	-8.7424248
N	-20.5799822	-32.4509759	-8.3489937
C	-17.9129840	-28.8779769	-6.3399942
C	-19.0050072	-27.8637909	-6.4312340
N	-20.1937514	-28.1049158	-7.0901568
C	-20.9865520	-27.0254445	-6.9507984
N	-20.3755546	-26.0968873	-6.2280400
C	-19.1336753	-26.5990404	-5.8952503
C	-9.8599912	-19.9459824	-1.6139985
N	-11.0831798	-20.3636322	-0.9418385
C	-12.1527998	-20.9413551	-1.5441079
N	-13.2609852	-21.1731459	-0.8411584
N	-12.1160142	-21.2978253	-2.8286235
C	-18.9870875	-17.8576085	-8.5726454
O	-19.9063986	-18.9457916	-8.6382071
C	-19.5839825	-16.7449883	-7.7059938
Fe	-21.4003494	-24.4458620	-5.4529287
O	-14.2944667	-22.5866055	-3.9174250
C	-15.3250482	-22.6550985	-3.1983828
O	-15.4216927	-22.2335984	-2.0108365
C	-16.5950124	-23.3067400	-3.7881650
C	-17.8011395	-22.3982501	-3.5813551
C	-19.1658847	-22.9844966	-3.7998772
O	-19.4283937	-24.0942473	-4.2336128
C	-21.4780984	-22.2839862	-3.3288283
O	-22.0250100	-23.1107986	-4.1080478
O	-21.9958302	-21.6056291	-2.4573313
C	-22.5858158	-24.4003989	2.7436431
O	-23.1504869	-25.4506700	1.9808441
C	-23.6728857	-25.1507519	0.7552732
C	-24.5736697	-26.0798045	0.2066126
C	-25.1572904	-25.8349011	-1.0337852
C	-23.3676619	-23.9901196	0.0353985
C	-23.9554207	-23.7654243	-1.2158312
C	-24.8613852	-24.6732703	-1.7724110
C	-25.4968245	-24.4859423	-3.1331950
C	-25.2451773	-23.1597867	-3.8490019
C	-25.8304776	-23.2081772	-5.2604891
O	-26.6288547	-24.0514514	-5.6462893
N	-25.3927528	-22.2055826	-6.0825145
C	-24.4288368	-21.2130031	-5.8133435
C	-23.5757001	-20.8460275	-6.8728161
C	-22.6324325	-19.8374737	-6.7147370
C	-22.5057736	-19.1923574	-5.4762044
C	-23.3490424	-19.5432642	-4.4288453
C	-24.3236422	-20.5499617	-4.5680920
C	-25.2829904	-20.7158821	-3.4180898
O	-25.6130234	-19.7278689	-2.7547709
N	-25.7848588	-21.9652444	-3.1701162
C	-26.9302980	-22.1126800	-2.2808828
O	-20.7994097	-23.3344218	-6.4717269
O	-19.6391276	-20.6931689	-6.2884641
H	-26.7874709	-16.5700288	-1.3016522
H	-26.4510130	-18.2737082	-1.1602756
H	-25.2070539	-15.0740621	-11.2170808
H	-26.5790419	-15.4975070	-10.1505849
H	-25.0709118	-16.4461179	-10.0944288
H	-25.3399901	-13.4660149	-9.2809717
H	-23.8367109	-14.4038197	-9.2062401
H	-24.5370965	-16.7804450	-6.1661240
H	-23.3307918	-16.2529313	-7.3733598
H	-24.7354779	-17.2546670	-7.8721475
H	-18.9506772	-25.9042989	2.2649421
H	-18.8661737	-26.0718707	0.4749929
H	-20.4255313	-25.6896538	1.2525246
H	-19.3913501	-23.4421181	2.0217983

H	-17.9364102	-23.8510534	1.1021833
H	-20.7187633	-21.8806411	-0.8819533
H	-20.3411917	-21.7791492	0.8208324
H	-20.5956212	-29.5660545	2.0543515
H	-20.5187448	-31.1676129	-0.4433845
H	-20.1495543	-31.6551407	1.1914775
H	-24.4733340	-29.0776270	-3.3359850
H	-24.4006391	-28.6411490	-1.6147705
H	-23.1679985	-29.7282247	-2.3185593
H	-20.6518747	-25.3856003	-2.3601048
H	-23.5835928	-26.9467847	-4.9656897
H	-24.8137784	-24.2994959	-9.2068999
H	-25.4503539	-24.7602810	-7.5706169
H	-24.6501111	-25.9724757	-8.6278336
H	-20.6897908	-22.1167747	-12.7882922
H	-21.3771760	-23.3507094	-11.6099577
H	-19.6042745	-23.2672915	-11.8929677
H	-21.1527299	-23.0578198	-9.2628233
H	-20.3551806	-21.6593829	-8.6398788
H	-14.7485819	-21.9309608	-10.5097950
H	-14.2770377	-21.7589403	-8.7989411
H	-15.0475838	-20.3853603	-9.6502883
H	-17.1960092	-21.5294247	-9.7481808
H	-15.8731553	-23.9402780	-8.3648915
H	-16.4151909	-23.8942404	-10.0631722
H	-17.6058794	-23.7682491	-8.7422373
H	-16.0069235	-21.7759179	-6.9181185
H	-17.7391923	-21.5889062	-7.3074902
H	-16.6114889	-20.2723276	-7.6551674
H	-18.8434627	-28.2219021	-11.8377347
H	-18.0165129	-27.1855484	-13.0248923
H	-19.6982398	-25.8680955	-11.9324421
H	-18.1382705	-25.2653579	-11.3695411
H	-19.9112369	-27.2284221	-9.7701744
H	-19.8772272	-25.4719661	-9.5013150
H	-18.4354168	-26.4465989	-9.1706704
H	-16.8995171	-27.9685231	-10.2344491
H	-16.0842752	-26.8809303	-11.3793395
H	-16.3335261	-28.5946363	-11.8000510
H	-21.2053087	-31.6727203	-10.1310539
H	-20.2857127	-32.3258266	-7.3839712
H	-20.6330403	-33.3932895	-8.7198670
H	-18.2588671	-29.7926772	-5.8282217
H	-17.0753012	-28.4559270	-5.7534374
H	-17.5093561	-29.1856608	-7.3292339
H	-21.9865403	-26.9451855	-7.3627183
H	-18.4415671	-26.0249529	-5.2863143
H	-10.0595822	-19.1386416	-2.3501373
H	-9.1531290	-19.5755643	-0.8614178
H	-9.3891294	-20.8044092	-2.1394744
H	-11.2277338	-20.0308281	0.0040399
H	-14.1229903	-21.5972494	-1.3054068
H	-13.2976489	-20.9096905	0.1356675
H	-11.2951036	-21.1174990	-3.3909315
H	-18.7565587	-17.4859630	-9.5891796
H	-18.0294550	-18.1968975	-8.1289990
H	-19.7461741	-19.4885093	-9.4489016
H	-20.3909303	-16.2010855	-8.2370597
H	-18.8210918	-16.0292697	-7.3655465
H	-20.0371914	-17.2640692	-6.8364081
H	-12.9440496	-21.7925282	-3.2646490
H	-16.4368328	-23.5448554	-4.8497487
H	-16.7667473	-24.2515533	-3.2441479
H	-17.7914518	-22.0117504	-2.5505598
H	-17.7660697	-21.5137157	-4.2433257
H	-21.6869820	-27.4429420	-1.2198952
H	-22.3466083	-24.8193323	3.7309922
H	-23.2958239	-23.5619946	2.8631914
H	-21.6574149	-24.0105543	2.2943671
H	-24.8054981	-26.9823602	0.7771215
H	-25.8626990	-26.5643999	-1.4436790
H	-22.6564939	-23.2585037	0.4170581
H	-23.6765652	-22.8576287	-1.7481476
H	-25.0969557	-25.2567267	-3.8112703
H	-26.5771996	-24.6912232	-3.0830141
H	-24.1574318	-23.0301816	-3.9488322
H	-25.7056905	-22.2892011	-7.0463221

H	-23.6520991	-21.3802572	-7.8236027
H	-21.9812420	-19.5560476	-7.5456069
H	-21.7447545	-18.4221212	-5.3359309
H	-23.2777743	-19.0400391	-3.4638863
H	-27.7479746	-22.6408334	-2.7971777
H	-27.2737689	-21.1146782	-1.9877514
H	-26.6630574	-22.6758823	-1.3736039
H	-20.2344247	-21.4604535	-6.3494458
O	-20.0843266	-22.0689968	-3.4514356
H	-27.5996472	-16.3675188	0.8213387
H	-19.8016103	-20.1379519	-7.0751003
H	-20.4271821	-28.9514157	-7.6307603

Supplementary Table 6. Coordinates of the QM cluster model of state 3 of the first reaction sequence, optimized with quintet ferryl.

C	-27.2579613	-17.3879751	0.5119999
O	-27.2618853	-18.3302800	1.2920753
N	-26.7989615	-17.4109751	-0.7359998
C	-25.4809628	-15.4299797	-10.2089851
C	-24.8861356	-14.4898210	-9.1642732
S	-25.3311843	-14.8930976	-7.4305371
C	-24.4029641	-16.4449784	-7.2049900
C	-19.3309706	-25.5119618	1.3099996
C	-18.9608642	-24.0442899	1.0710579
C	-19.2502051	-23.6466820	-0.3783280
O	-19.0714347	-24.4530792	-1.2945866
N	-19.6996788	-22.3956144	-0.5995381
C	-20.7219712	-29.7009597	0.9539997
O	-21.1039513	-28.7836491	0.2350386
N	-20.4449713	-30.9339533	0.5429998
C	-23.7809663	-28.8239609	-2.5189953
C	-22.9543212	-27.6321838	-2.8878619
N	-21.9617912	-27.0976668	-2.0818685
C	-21.4384246	-26.0108736	-2.6969427
N	-22.0423762	-25.8184994	-3.8644925
C	-22.9836338	-26.8226321	-4.0011343
C	-24.6379651	-24.9089650	-8.3089877
C	-23.2666906	-24.5752504	-7.7069323
O	-23.0070399	-25.1144839	-6.5509088
O	-22.4642670	-23.8669453	-8.3148193
C	-20.5389712	-22.6719696	-11.8559827
C	-20.2950327	-21.6497857	-10.7413895
O	-19.6163681	-20.6309674	-10.9612490
N	-20.7815043	-21.9478829	-9.5306505
C	-15.0539798	-21.4899710	-9.5369853
C	-16.4530832	-21.9578005	-9.0977854
C	-16.5800429	-23.4863101	-9.1211999
C	-16.8113880	-21.4031555	-7.7105122
C	-18.1399729	-27.3639623	-11.9339826
C	-18.8204894	-26.1264280	-11.3202189
C	-19.2827219	-26.3027565	-9.8708852
C	-16.7942101	-27.7226554	-11.3021951
C	-20.8929711	-31.3979572	-9.0979884
O	-20.8373201	-30.2210684	-8.7572499
N	-20.5799712	-32.4509609	-8.3489877
C	-17.9129740	-28.8779619	-6.3399902
C	-19.0195719	-27.8842303	-6.4368834
N	-20.2022167	-28.1722059	-7.0891556
C	-21.0272355	-27.1210974	-6.9680499
N	-20.4427536	-26.1575591	-6.2631942
C	-19.1834129	-26.6165115	-5.9238927
C	-9.8599852	-19.9459704	-1.6139985
N	-11.1304514	-20.3702037	-1.0008336
C	-12.2153925	-20.8944782	-1.6345471
N	-13.3704536	-21.0259296	-0.9782243
N	-12.1675743	-21.3019210	-2.9063100
C	-19.0275885	-17.8108154	-8.6490181
O	-20.1020784	-18.6231858	-9.0731125
C	-19.5839715	-16.7449773	-7.7059898
Fe	-21.4330134	-24.5309434	-5.4770313
O	-14.5228165	-22.6219977	-3.9504709
C	-15.5584736	-22.6106975	-3.2324709
O	-15.6560597	-22.0260003	-2.1141627
C	-16.8241113	-23.3773876	-3.7222328

C	-18.0243445	-22.4329453	-3.9092320
C	-19.4585382	-22.9822676	-3.9930227
O	-19.6899149	-23.9910988	-4.7937418
C	-20.1466340	-19.6885966	-3.2887136
O	-19.5979437	-19.6421100	-4.3149851
O	-20.6790930	-19.6694872	-2.2557068
C	-22.4671347	-23.9408312	2.6094982
O	-23.0419449	-24.9978547	1.8618380
C	-23.4571904	-24.7350429	0.5866539
C	-24.5026495	-25.5280699	0.0902531
C	-25.0015922	-25.2945815	-1.1885530
C	-22.9008026	-23.7417388	-0.2299602
C	-23.3944952	-23.5383309	-1.5246269
C	-24.4704496	-24.2897120	-2.0168997
C	-25.0590222	-24.0932375	-3.3940885
C	-24.9212773	-22.7073919	-4.0134383
C	-25.5455997	-22.7015714	-5.4089885
O	-26.3568156	-23.5267138	-5.8055131
N	-25.1294680	-21.6606066	-6.1907844
C	-24.2031400	-20.6541184	-5.8557319
C	-23.3247127	-20.2298747	-6.8677174
C	-22.4144120	-19.2028768	-6.6456983
C	-22.3761794	-18.5755913	-5.3924183
C	-23.2391005	-18.9905306	-4.3823136
C	-24.1575755	-20.0386125	-4.5796536
C	-25.1157504	-20.3102276	-3.4468560
O	-25.5064425	-19.3741790	-2.7413382
N	-25.5231442	-21.6000267	-3.2492024
C	-26.6537863	-21.8762150	-2.3710653
O	-22.1100024	-23.0572033	-5.2645144
O	-20.2240113	-21.4293841	-6.7386043
H	-26.8372776	-16.5796920	-1.3147876
H	-26.3928371	-18.2526289	-1.1520828
H	-25.2033917	-15.1013823	-11.2253289
H	-26.5802297	-15.4513548	-10.1385071
H	-25.1113973	-16.4601617	-10.0824498
H	-25.2511942	-13.4602320	-9.3112343
H	-23.7856279	-14.4567215	-9.2401411
H	-24.5276009	-16.7545660	-6.1578157
H	-23.3284537	-16.2967591	-7.3959547
H	-24.7753303	-17.2550782	-7.8498246
H	-18.9559357	-25.8848159	2.2744554
H	-18.8661617	-26.0718557	0.4749929
H	-20.4255203	-25.6896388	1.2525246
H	-19.4582725	-23.3560009	1.7735971
H	-17.8743876	-23.9069072	1.2198288
H	-19.9546025	-22.1439345	-1.5630744
H	-19.8441716	-21.7383578	0.1558982
H	-20.5735266	-29.5603082	2.0505806
H	-20.5347906	-31.1728042	-0.4408408
H	-20.1286492	-31.6486507	1.1887917
H	-24.4662788	-29.0618349	-3.3457379
H	-24.4006261	-28.6411340	-1.6147705
H	-23.1679855	-29.7282077	-2.3185573
H	-20.6285477	-25.4032099	-2.2877834
H	-23.6117013	-26.8823366	-4.8858371
H	-24.8031340	-24.2768529	-9.1915535
H	-25.4503389	-24.7602680	-7.5706129
H	-24.6500961	-25.9724597	-8.6278286
H	-20.6885209	-22.1662106	-12.8182635
H	-21.3771650	-23.3506974	-11.6099517
H	-19.6042635	-23.2672795	-11.8929597
H	-21.3562450	-22.7676844	-9.3338636
H	-20.5125023	-21.4033630	-8.7035001
H	-14.7485739	-21.9309498	-10.5097890
H	-14.2899510	-21.7656232	-8.7884867
H	-15.0475758	-20.3853493	-9.6502843
H	-17.1876113	-21.5485287	-9.8176627
H	-15.8752645	-23.9507304	-8.4080469
H	-16.3622751	-23.8961105	-10.1225651
H	-17.5969796	-23.8054086	-8.8393952
H	-16.1308125	-21.8029082	-6.9359825
H	-17.8434482	-21.6652446	-7.4273623
H	-16.7283503	-20.3025785	-7.6891206
H	-18.8434507	-28.2218871	-11.8377277
H	-18.0165029	-27.1855334	-13.0248843
H	-19.6940038	-25.8633618	-11.9429834

H	-18.1324302	-25.2628577	-11.3842461
H	-19.9247904	-27.1939451	-9.7669816
H	-19.8607722	-25.4323756	-9.5196181
H	-18.4369145	-26.4278635	-9.1757111
H	-16.9017511	-27.9647021	-10.2316591
H	-16.0847072	-26.8805847	-11.3786383
H	-16.3345809	-28.5953426	-11.7949329
H	-21.2317852	-31.6764429	-10.1215269
H	-20.2606356	-32.3266709	-7.3917868
H	-20.6555387	-33.3940720	-8.7139477
H	-18.2487232	-29.7966151	-5.8280128
H	-17.0752912	-28.4559110	-5.7534354
H	-17.5093471	-29.1856458	-7.3292299
H	-22.0346312	-27.0798903	-7.3682725
H	-18.5114703	-26.0014553	-5.3323472
H	-10.0595762	-19.1386306	-2.3501353
H	-9.1878804	-19.5885152	-0.8259722
H	-9.3891254	-20.8043972	-2.1394724
H	-11.3037448	-20.0335227	-0.0606468
H	-14.2436027	-21.4150717	-1.4517009
H	-13.4491730	-20.6821792	-0.0288676
H	-11.3015986	-21.2819228	-3.4285706
H	-18.5256206	-17.3219676	-9.5112659
H	-18.2485235	-18.4045187	-8.1257328
H	-19.7962983	-19.3021591	-9.7183407
H	-20.3909183	-16.2010775	-8.2370557
H	-18.8123796	-16.0408156	-7.3604606
H	-20.0371804	-17.2640602	-6.8364041
H	-13.0100099	-21.7670854	-3.3273219
H	-16.5868086	-23.9182821	-4.6503133
H	-17.0819825	-24.1067852	-2.9366262
H	-18.0259062	-21.7330438	-3.0622446
H	-17.8953004	-21.8351374	-4.8288450
H	-21.6542482	-27.4972530	-1.1899483
H	-22.3920968	-24.2913237	3.6485632
H	-23.0987824	-23.0349744	2.5729220
H	-21.4564929	-23.6802814	2.2570549
H	-24.9239112	-26.3033816	0.7344276
H	-25.8349946	-25.9023403	-1.5541138
H	-22.0644609	-23.1357403	0.1153532
H	-22.9049400	-22.7907866	-2.1536175
H	-24.5534738	-24.7645469	-4.1057416
H	-26.1133630	-24.4058397	-3.4031605
H	-23.8484884	-22.5002831	-4.1368518
H	-25.4442556	-21.6978268	-7.1571583
H	-23.3367498	-20.7526258	-7.8275262
H	-21.7249825	-18.9170218	-7.4449711
H	-21.6722693	-17.7614607	-5.2067090
H	-23.2304495	-18.5020270	-3.4062779
H	-27.4362769	-22.4222596	-2.9224044
H	-27.0616251	-20.9241802	-2.0142089
H	-26.3459795	-22.4810448	-1.5048356
H	-20.9532870	-21.8731978	-6.2610677
O	-20.3400348	-22.4086697	-3.3494102
H	-27.6608282	-16.3836683	0.8000480
H	-19.9943570	-20.6848467	-6.1660738
H	-20.4100277	-29.0302695	-7.6246140

Supplementary Table 7. Coordinates of the QM cluster model of state 4 of the first reaction sequence, optimized with quintet ferryl.

C	-27.2579533	-17.3879711	0.5119999
O	-27.2272640	-18.3204547	1.3026368
N	-26.7989545	-17.4109701	-0.7359998
C	-25.4809558	-15.4299747	-10.2089821
C	-24.9521367	-14.4804277	-9.1376515
S	-25.4047923	-14.9380644	-7.4194589
C	-24.4029571	-16.4449744	-7.2049880
C	-19.3309656	-25.5119538	1.3099996
C	-18.9377805	-24.0520996	1.1093452
C	-19.0972024	-23.6521250	-0.3575318
O	-18.9749692	-24.4898133	-1.2542316
N	-19.3564314	-22.3556816	-0.6185346
C	-20.7219662	-29.7009517	0.9539997
O	-21.1266135	-28.7889147	0.2402305

N	-20.4449653	-30.9339453	0.5429998
C	-23.7809593	-28.8239529	-2.5189943
C	-22.9500083	-27.6304573	-2.8918772
N	-21.9380265	-27.1133248	-2.0967741
C	-21.4061782	-26.0308256	-2.7147057
N	-22.0224671	-25.8205055	-3.8725151
C	-22.9835815	-26.8076263	-3.9972880
C	-24.6379571	-24.9089570	-8.3089857
C	-23.2486471	-24.6213843	-7.7246152
O	-23.0709059	-24.9634126	-6.4789372
O	-22.3626753	-24.1250672	-8.4235080
C	-20.5389652	-22.6719636	-11.8559797
C	-20.3056898	-21.6630347	-10.7266132
O	-19.6466099	-20.6290051	-10.9358822
N	-20.7838585	-21.9868580	-9.5179917
C	-15.0539758	-21.4899650	-9.5369823
C	-16.4591806	-21.9436783	-9.1052081
C	-16.5991488	-23.4709456	-9.0976150
C	-16.8254491	-21.3563729	-7.7348260
C	-18.1399679	-27.3639543	-11.9339796
C	-18.8242309	-26.1312263	-11.3145827
C	-19.2880585	-26.3195948	-9.8668528
C	-16.7937670	-27.7219910	-11.3027146
C	-20.8929651	-31.3979482	-9.0979854
O	-20.8705058	-30.2236226	-8.7437390
N	-20.5799652	-32.4509529	-8.3489857
C	-17.9129680	-28.8779539	-6.3399882
C	-19.0244914	-27.8802209	-6.4389642
N	-20.2208516	-28.1746218	-7.0681919
C	-21.0425220	-27.1190573	-6.9501619
N	-20.4453271	-26.1456978	-6.2711491
C	-19.1794982	-26.6024609	-5.9457564
C	-9.8599822	-19.9459654	-1.6139985
N	-11.1302466	-20.3637615	-0.9995442
C	-12.2243803	-20.8517071	-1.6468751
N	-13.3891530	-20.9513554	-1.0035020
N	-12.1729685	-21.2525903	-2.9203176
C	-19.0371680	-17.8118461	-8.6537496
O	-20.1229468	-18.5982338	-9.0967534
C	-19.5839655	-16.7449733	-7.7059878
Fe	-21.3939301	-24.5344529	-5.5065038
O	-14.4958644	-22.5706529	-3.9431046
C	-15.5496636	-22.5133944	-3.2530915
O	-15.6737517	-21.8577689	-2.1775478
C	-16.7986823	-23.3149614	-3.7158658
C	-17.9869272	-22.3984699	-4.0817771
C	-19.4005957	-22.9991690	-4.0326688
O	-19.6609457	-23.9615911	-4.8741506
C	-20.5435976	-19.9134018	-3.1809063
O	-19.8565633	-19.7160358	-4.1006966
O	-21.2284868	-20.0291375	-2.2483812
C	-22.2242363	-22.8087109	1.8658222
O	-22.5682695	-24.0584961	1.2904173
C	-23.1773367	-24.0271864	0.0597920
C	-24.3001702	-24.8429656	-0.1220798
C	-24.9678786	-24.8369537	-1.3483072
C	-22.7042454	-23.2461492	-1.0037904
C	-23.3655887	-23.2663415	-2.2310717
C	-24.5257719	-24.0354824	-2.4099227
C	-25.2857227	-23.9952921	-3.7143663
C	-25.5198033	-22.6149497	-4.2664933
C	-25.9071516	-22.5468160	-5.6852517
O	-26.5463528	-23.4627184	-6.2009105
N	-25.5263061	-21.4481255	-6.4191098
C	-24.4818460	-20.5557278	-6.1037543
C	-23.5823526	-20.2111630	-7.1285862
C	-22.5495034	-19.3044379	-6.9051198
C	-22.4165766	-18.7143780	-5.6420543
C	-23.2984738	-19.0529568	-4.6210637
C	-24.3289877	-19.9899122	-4.8202379
C	-25.2192017	-20.2382251	-3.6416665
O	-25.4432755	-19.3438463	-2.8266326
N	-25.7213346	-21.5094872	-3.4282523
C	-26.4736786	-21.6838534	-2.1750401
O	-22.0089168	-22.8170647	-5.4011378
O	-20.1830034	-21.3131556	-6.7809606
H	-26.8659367	-16.5908719	-1.3280478

H	-26.3716632	-18.2466005	-1.1390657
H	-25.2033704	-15.0691532	-11.2143008
H	-26.5787499	-15.5079413	-10.1583225
H	-25.0618364	-16.4424598	-10.0944102
H	-25.3668045	-13.4681384	-9.2726370
H	-23.8536722	-14.3898337	-9.1937593
H	-24.5393123	-16.7854385	-6.1690547
H	-23.3328935	-16.2385692	-7.3642528
H	-24.7169313	-17.2578145	-7.8764690
H	-18.9654219	-25.9072191	2.2704894
H	-18.8661557	-26.0718487	0.4749929
H	-20.4255143	-25.6896308	1.2525246
H	-19.4922494	-23.3650956	1.7660312
H	-17.8672948	-23.9172227	1.3522102
H	-19.5896893	-22.1258372	-1.5940046
H	-19.4654518	-21.6732927	0.1196938
H	-20.5512252	-29.5544546	2.0464258
H	-20.5523073	-31.1781452	-0.4377948
H	-20.1073421	-31.6421539	1.1851358
H	-24.4668277	-29.0615967	-3.3454140
H	-24.4006181	-28.6411260	-1.6147705
H	-23.1679795	-29.7282007	-2.3185563
H	-20.5883426	-25.4316677	-2.3081083
H	-23.6293360	-26.8485140	-4.8706669
H	-24.7945471	-24.2800779	-9.1955107
H	-25.4503309	-24.7602600	-7.5706109
H	-24.6500901	-25.9724527	-8.6278266
H	-20.6882042	-22.1570987	-12.8131722
H	-21.3771590	-23.3506904	-11.6099487
H	-19.6042575	-23.2672725	-11.8929567
H	-21.3218897	-22.8351600	-9.3358118
H	-20.5186773	-21.4495095	-8.6814330
H	-14.7485699	-21.9309438	-10.5097860
H	-14.2952727	-21.7677560	-8.7841434
H	-15.0475718	-20.3853433	-9.6502823
H	-17.1835565	-21.5440636	-9.8407435
H	-15.9102241	-23.9253031	-8.3628580
H	-16.3698087	-23.9050214	-10.0861386
H	-17.6233078	-23.7740144	-8.8241900
H	-16.1621402	-21.7553762	-6.9452919
H	-17.8667072	-21.5888304	-7.4613217
H	-16.7176797	-20.2576633	-7.7329483
H	-18.8434457	-28.2218801	-11.8377247
H	-18.0164969	-27.1855264	-13.0248803
H	-19.6975956	-25.8672693	-11.9371425
H	-18.1392987	-25.2646300	-11.3720542
H	-19.9181035	-27.2202443	-9.7685347
H	-19.8803886	-25.4604116	-9.5124417
H	-18.4421714	-26.4365546	-9.1702639
H	-16.9009979	-27.9650562	-10.2324007
H	-16.0852386	-26.8789935	-11.3783626
H	-16.3329684	-28.5935750	-11.7963943
H	-21.1976220	-31.6728918	-10.1330622
H	-20.2902890	-32.3280586	-7.3822364
H	-20.6254403	-33.3923125	-8.7233569
H	-18.2523711	-29.7945011	-5.8272924
H	-17.0752862	-28.4559040	-5.7534344
H	-17.5093431	-29.1856378	-7.3292279
H	-22.0561893	-27.0828733	-7.3364494
H	-18.4980346	-25.9765226	-5.3772184
H	-10.0595732	-19.1386246	-2.3501343
H	-9.1857816	-19.5885791	-0.8276206
H	-9.3891234	-20.8043922	-2.1394714
H	-11.3040267	-20.0200304	-0.0619663
H	-14.2719819	-21.3020931	-1.4934971
H	-13.4682688	-20.6014413	-0.0564094
H	-11.2955401	-21.2715084	-3.4232666
H	-18.5176672	-17.3238874	-9.5063368
H	-18.2751337	-18.4260879	-8.1298543
H	-19.8210413	-19.3008329	-9.7190215
H	-20.3909133	-16.2010735	-8.2370537
H	-18.8092861	-16.0438839	-7.3616562
H	-20.0371744	-17.2640562	-6.8364021
H	-13.0185293	-21.7148928	-3.3444390
H	-16.5236011	-23.9624846	-4.5618028
H	-17.1014257	-23.9472307	-2.8650939
H	-17.9900925	-21.5574273	-3.3755571

H	-17.8552904	-21.9898703	-5.0960838
H	-21.6239223	-27.5213525	-1.2111767
H	-21.8404208	-23.0162249	2.8749154
H	-23.1067329	-22.1471721	1.9410021
H	-21.4424351	-22.2837802	1.2933703
H	-24.6468655	-25.4555983	0.7133818
H	-25.8624664	-25.4545481	-1.4750336
H	-21.8004152	-22.6494620	-0.9009688
H	-22.9430506	-22.6947138	-3.0617513
H	-24.7258899	-24.5420284	-4.4952181
H	-26.2438829	-24.5404628	-3.6178604
H	-22.8932116	-22.7540831	-5.7901061
H	-25.7944324	-21.5060901	-7.3988112
H	-23.6849149	-20.6885866	-8.1067844
H	-21.8429012	-19.0671256	-7.7058457
H	-21.6193041	-17.9934523	-5.4533877
H	-23.2106330	-18.5986330	-3.6329812
H	-26.9568483	-22.6654573	-2.1728309
H	-27.2394449	-20.8985892	-2.1047971
H	-25.8056969	-21.6033391	-1.3053154
H	-20.8584655	-21.8052144	-6.2531868
O	-20.2256394	-22.5394303	-3.2405999
H	-27.6946236	-16.3947287	0.7878158
H	-20.0006299	-20.5255619	-6.2528167
H	-20.4373948	-29.0355171	-7.5956502

Supplementary Table 8. Coordinates of the QM cluster model of state 5 of the first reaction sequence, optimized with quintet ferryl.

C	-27.2579610	-17.3879760	0.5120000
O	-27.0970759	-18.2693635	1.3471882
N	-26.7989620	-17.4109750	-0.7360000
C	-25.4809630	-15.4299790	-10.2089850
C	-25.0143989	-14.4730684	-9.1158443
S	-25.4692047	-14.9811350	-7.4129868
C	-24.4029640	-16.4449790	-7.2049900
C	-19.3309710	-25.5119610	1.3100000
C	-19.0582378	-24.0269418	1.0413349
C	-19.3391183	-23.6822306	-0.4258344
O	-18.9983866	-24.4530770	-1.3261836
N	-19.9372784	-22.5010569	-0.6783436
C	-20.7219720	-29.7009600	0.9540000
O	-21.0849901	-28.7703149	0.2409049
N	-20.4449710	-30.9339540	0.5430000
C	-23.7809660	-28.8239610	-2.5189950
C	-22.9172155	-27.6528020	-2.8724970
N	-21.7972471	-27.2824770	-2.1423740
C	-21.2451169	-26.1889662	-2.7232497
N	-21.9483812	-25.8354521	-3.7925244
C	-22.9916944	-26.7370177	-3.8999204
C	-24.6379640	-24.9089640	-8.3089880
C	-23.3323582	-24.5351523	-7.6072654
O	-23.1449996	-25.0843330	-6.4701063
O	-22.5479460	-23.7223993	-8.1353971
C	-20.5389710	-22.6719700	-11.8559830
C	-20.2823201	-21.7370495	-10.6707173
O	-19.6312831	-20.6846005	-10.8088315
N	-20.7040158	-22.1748558	-9.4769119
C	-15.0539800	-21.4899710	-9.5369850
C	-16.4505706	-21.9418420	-9.0772550
C	-16.5859156	-23.4689980	-9.0438132
C	-16.7876942	-21.3315239	-7.7083197
C	-18.1399730	-27.3639620	-11.9339830
C	-18.8155950	-26.1195866	-11.3289536
C	-19.2842226	-26.2813026	-9.8810686
C	-16.7955961	-27.7229532	-11.2995438
C	-20.8929710	-31.3979570	-9.0979880
O	-20.8842233	-30.2254779	-8.7402018
N	-20.5799710	-32.4509620	-8.3489880
C	-17.9129730	-28.8779620	-6.3399900
C	-18.9940681	-27.8674938	-6.4630306
N	-20.1872671	-28.1390868	-7.1012862
C	-20.9679354	-27.0453734	-7.0197351
N	-20.3425769	-26.0798765	-6.3611627
C	-19.1058167	-26.5739511	-6.0055197

C	-9.8599850	-19.9459710	-1.6139990
N	-11.0848987	-20.3631211	-0.9421657
C	-12.1665530	-20.9256580	-1.5391664
N	-13.2477044	-21.2143483	-0.8153420
N	-12.1717665	-21.2054404	-2.8433150
C	-18.9597663	-17.8282573	-8.5876885
O	-19.8688167	-18.9196312	-8.6991601
C	-19.5839710	-16.7449780	-7.7059900
Fe	-21.3989396	-24.5450975	-5.4174998
O	-14.3937009	-22.4175072	-3.9197966
C	-15.3763109	-22.6158686	-3.1536624
O	-15.4101242	-22.3158533	-1.9244559
C	-16.6545771	-23.2494038	-3.7340216
C	-17.8498067	-22.3234271	-3.5245248
C	-19.2376895	-22.8092059	-3.9282970
O	-19.4076462	-23.8388000	-4.6222067
C	-20.1323113	-19.4784538	-3.2164519
O	-19.5002427	-19.3141017	-4.1832660
O	-20.7604597	-19.5446282	-2.2406170
C	-22.0091711	-22.6155134	3.2868097
O	-22.2361078	-23.7405230	2.4551594
C	-22.7312800	-23.5540859	1.2064370
C	-22.9891786	-24.7176534	0.4592103
C	-23.4810917	-24.6164659	-0.8339719
C	-22.9711311	-22.2961216	0.6266859
C	-23.4627585	-22.2103255	-0.6772542
C	-23.7458516	-23.3641217	-1.4330929
C	-24.2631815	-23.3576486	-2.8002909
C	-24.9274679	-22.3962714	-3.4748016
C	-25.3294413	-22.6704062	-4.8921136
O	-25.8191824	-23.7294236	-5.2517498
N	-25.1261979	-21.6413346	-5.7878574
C	-24.2374240	-20.5625870	-5.6581333
C	-23.4828274	-20.2110848	-6.7937089
C	-22.5682501	-19.1667009	-6.7513367
C	-22.3854061	-18.4448104	-5.5638092
C	-23.1516771	-18.7579595	-4.4496958
C	-24.0846278	-19.8123979	-4.4704488
C	-24.9469877	-19.9437488	-3.2580206
O	-25.2412605	-18.9455317	-2.5925092
N	-25.3987439	-21.1786354	-2.8932314
C	-26.3657902	-21.2347017	-1.7909952
O	-21.9805045	-22.5547323	-5.2842890
O	-20.1242738	-20.9311478	-6.7905433
H	-26.9801450	-16.6318039	-1.3590940
H	-26.2495132	-18.1857752	-1.1193576
H	-25.2089179	-15.0400583	-11.2049806
H	-26.5740150	-15.5631572	-10.1746037
H	-25.0141447	-16.4224349	-10.1038791
H	-25.4766353	-13.4803455	-9.2412298
H	-23.9210209	-14.3271659	-9.1545357
H	-24.5489007	-16.8082842	-6.1789973
H	-23.3408079	-16.1849447	-7.3340695
H	-24.6651476	-17.2585507	-7.8972841
H	-18.9443339	-25.8518534	2.2811083
H	-18.8661610	-26.0718560	0.4749930
H	-20.4255200	-25.6896380	1.2525250
H	-19.6414139	-23.3801486	1.7151463
H	-17.9911088	-23.7941546	1.2098598
H	-20.1722834	-22.2540828	-1.6486341
H	-20.2989591	-21.9249621	0.0691258
H	-20.5969013	-29.5683710	2.0536685
H	-20.5114875	-31.1699931	-0.4435636
H	-20.1424750	-31.6505922	1.1933237
H	-24.4643295	-29.0490836	-3.3503747
H	-24.4006250	-28.6411340	-1.6147710
H	-23.1679860	-29.7282090	-2.3185570
H	-20.3596419	-25.6695989	-2.3494923
H	-23.7032664	-26.6520193	-4.7183397
H	-24.8015853	-24.2751335	-9.1922434
H	-25.4503380	-24.7602670	-7.5706130
H	-24.6500970	-25.9724600	-8.6278290
H	-20.6870967	-22.1195714	-12.7909962
H	-21.3771650	-23.3506970	-11.6099520
H	-19.6042630	-23.2672790	-11.8929600
H	-21.3526446	-22.9515090	-9.3195607
H	-20.4265707	-21.6912727	-8.6241467

H	-14.7485740	-21.9309500	-10.5097890
H	-14.2864166	-21.7623623	-8.7908637
H	-15.0475760	-20.3853490	-9.6502850
H	-17.1903807	-21.5537799	-9.8034935
H	-15.8803396	-23.9105153	-8.3172459
H	-16.3753292	-23.9161847	-10.0305519
H	-17.6031378	-23.7722102	-8.7461246
H	-16.1025340	-21.7075377	-6.9264072
H	-17.8185374	-21.5654524	-7.4019802
H	-16.6931418	-20.2317127	-7.7318156
H	-18.8434510	-28.2218880	-11.8377280
H	-18.0165020	-27.1855340	-13.0248840
H	-19.6846622	-25.8549615	-11.9575430
H	-18.1207210	-25.2613827	-11.3979547
H	-19.9429062	-27.1594686	-9.7752673
H	-19.8458050	-25.3990546	-9.5338304
H	-18.4441562	-26.4190347	-9.1815535
H	-16.9051117	-27.9567904	-10.2274028
H	-16.0827641	-26.8841506	-11.3814983
H	-16.3389106	-28.6008811	-11.7857019
H	-21.1838399	-31.6739161	-10.1373118
H	-20.3034305	-32.3283535	-7.3783823
H	-20.6159401	-33.3915320	-8.7260971
H	-18.2624348	-29.7910047	-5.8273934
H	-17.0752910	-28.4559120	-5.7534360
H	-17.5093480	-29.1856460	-7.3292300
H	-21.9820936	-26.9791696	-7.4022014
H	-18.4116963	-25.9642699	-5.4321247
H	-10.0595760	-19.1386300	-2.3501350
H	-9.1552338	-19.5722400	-0.8612559
H	-9.3891260	-20.8043980	-2.1394720
H	-11.1838586	-20.1248341	0.0371582
H	-14.1141772	-21.6553257	-1.2681248
H	-13.2466329	-21.0257680	0.1793816
H	-11.3743392	-20.9794510	-3.4226097
H	-18.7160574	-17.4261418	-9.5889343
H	-18.0066054	-18.1700667	-8.1370172
H	-19.6660498	-19.4895646	-9.4860605
H	-20.3909190	-16.2010780	-8.2370560
H	-18.8330262	-16.0215828	-7.3546842
H	-20.0371800	-17.2640610	-6.8364040
H	-13.0198691	-21.6716608	-3.2786550
H	-16.5085219	-23.4800590	-4.7991523
H	-16.8532388	-24.1894282	-3.1926511
H	-17.9044772	-22.0446785	-2.4619143
H	-17.6998922	-21.3743524	-4.0722283
H	-21.4551929	-27.7675643	-1.3045519
H	-21.6446450	-23.0061161	4.2461578
H	-22.9368702	-22.0409159	3.4556221
H	-21.2436286	-21.9407091	2.8630275
H	-22.7911669	-25.6926328	0.9095889
H	-23.6783508	-25.5261400	-1.4040368
H	-22.7653715	-21.3757780	1.1739269
H	-23.5958363	-21.2212688	-1.1157312
H	-21.3894682	-22.2449680	-4.5154940
H	-24.1006427	-24.2777187	-3.3670495
H	-21.6731525	-22.0293684	-6.0500637
H	-25.3347839	-21.9213975	-6.7434784
H	-23.5775261	-20.8202229	-7.6967100
H	-21.9713223	-18.9327511	-7.6344991
H	-21.6631744	-17.6283187	-5.5172501
H	-23.0576438	-18.1821713	-3.5275807
H	-26.7097603	-22.2691024	-1.6768199
H	-27.2210150	-20.5804277	-2.0175948
H	-25.9246363	-20.8914642	-0.8423444
H	-19.7232881	-20.7371223	-5.9313219
O	-20.1938669	-22.0492965	-3.5410437
H	-27.8300333	-16.4541913	0.7448521
H	-19.9984953	-20.1137738	-7.3262705
H	-20.4255127	-29.0044086	-7.6057960

Supplementary Table 9. Coordinates of the QM cluster model of state 1 of the second reaction sequence, optimized with quintet ferryl.

C	-27.2579763	-17.3879861	0.5119999
O	-27.2116800	-18.3198748	1.3053532
N	-26.7989775	-17.4109851	-0.7359998
C	-25.4809788	-15.4299877	-10.2089911
C	-24.9053662	-14.4869859	-9.1560865
S	-25.3538133	-14.9065207	-7.4271690
C	-24.4029781	-16.4449874	-7.2049940
C	-19.3309826	-25.5119768	1.3099996
C	-19.0575587	-24.0145561	1.1306372
C	-19.5828696	-23.5209369	-0.2168348
O	-19.3513489	-24.1509372	-1.2502888
N	-20.2881381	-22.3701553	-0.2071293
C	-20.7219832	-29.7009767	0.9539997
O	-21.0889540	-28.7737775	0.2358826
N	-20.4449823	-30.9339723	0.5429998
C	-23.7809803	-28.8239759	-2.5189973
C	-22.9383787	-27.6510031	-2.9318459
N	-21.8368925	-27.2169451	-2.2110383
C	-21.3120364	-26.1303512	-2.8260467
N	-22.0109505	-25.8433169	-3.9207438
C	-23.0276067	-26.7778974	-4.0006283
C	-24.6379781	-24.9089780	-8.3089927
C	-23.3593948	-24.3727290	-7.6854844
O	-22.9964755	-24.9392484	-6.5691300
O	-22.8089861	-23.4093496	-8.2184279
C	-20.5389822	-22.6719826	-11.8559897
C	-20.2745180	-21.6450301	-10.7478000
O	-19.6330575	-20.6120477	-11.0005011
N	-20.6759648	-21.9450881	-9.5012456
C	-15.0539878	-21.4899820	-9.5369913
C	-16.4379378	-21.9383346	-9.0440673
C	-16.6013780	-23.4629017	-9.0521856
C	-16.7009533	-21.3666424	-7.6438160
C	-18.1399849	-27.3639773	-11.9339886
C	-18.8016388	-26.1256202	-11.3029157
C	-19.2081520	-26.2935081	-9.8353089
C	-16.7891948	-27.7372249	-11.3186947
C	-20.8929821	-31.3979752	-9.0979934
O	-20.8872491	-30.2249241	-8.7387098
N	-20.5799822	-32.4509759	-8.3489937
C	-17.9129840	-28.8779769	-6.3399942
C	-19.0309257	-27.9116233	-6.4144166
N	-20.2257527	-28.1959577	-7.0402920
C	-21.0554965	-27.1505032	-6.8670976
N	-20.4488533	-26.2149541	-6.1521527
C	-19.1826249	-26.6617791	-5.8617030
C	-9.8599912	-19.9459824	-1.6139985
N	-11.0848783	-20.3601354	-0.9397140
C	-12.1661535	-20.9193070	-1.5401247
N	-13.2773739	-21.1324439	-0.8360738
N	-12.1391903	-21.2751312	-2.8253935
C	-19.0478763	-17.8206981	-8.6512421
O	-20.1464961	-18.5759284	-9.1237632
C	-19.5839825	-16.7449883	-7.7059938
Fe	-21.3672355	-24.5229197	-5.5044677
O	-14.3349414	-22.5795563	-3.8791964
C	-15.3640239	-22.6295497	-3.1562393
O	-15.4625909	-22.1617973	-1.9860799
C	-16.6292113	-23.3163619	-3.7156678
C	-17.8383011	-22.3966563	-3.5821174
C	-19.1992593	-22.9939028	-3.8041126
O	-19.4457176	-24.1059276	-4.2447179
C	-21.5524326	-22.2796833	-3.4900785
O	-22.0232654	-23.0185985	-4.3946152
O	-22.1232007	-21.6414916	-2.6285781
C	-22.2347096	-23.2095209	3.3187040
O	-22.4588436	-24.2820340	2.4223517
C	-23.0581868	-24.0375646	1.2287942
C	-23.2856283	-25.1560099	0.4083708
C	-23.8587012	-24.9852371	-0.8440217
C	-23.4253943	-22.7600799	0.7705550
C	-23.9803433	-22.6041853	-0.4995253
C	-24.2136206	-23.7101532	-1.3404358
C	-24.6796751	-23.6344652	-2.7199835
C	-25.1630199	-22.6073580	-3.4534655
C	-25.4006764	-22.8680476	-4.9075357
O	-25.8944718	-23.9105577	-5.3196434

N	-25.0201181	-21.8824066	-5.7901060
C	-24.1370776	-20.8056367	-5.6009335
C	-23.3075840	-20.4733304	-6.6925145
C	-22.4417205	-19.3845739	-6.6352875
C	-22.3627317	-18.6250365	-5.4569668
C	-23.1801658	-18.9439616	-4.3794136
C	-24.0838519	-20.0227130	-4.4257337
C	-25.0193280	-20.1348946	-3.2630417
O	-25.3224717	-19.1249138	-2.6185521
N	-25.5623702	-21.3441325	-2.9240726
C	-26.6306990	-21.3137827	-1.9158099
O	-20.6230520	-23.6356055	-6.6421172
O	-19.8048167	-21.0839239	-6.5463254
H	-26.8702665	-16.5862140	-1.3214664
H	-26.3354220	-18.2280060	-1.1444585
H	-25.2034850	-15.0913667	-11.2220724
H	-26.5801157	-15.4683378	-10.1439416
H	-25.0965710	-16.4551836	-10.0867026
H	-25.2845711	-13.4619739	-9.2993434
H	-23.8050675	-14.4374782	-9.2258195
H	-24.5371984	-16.7674614	-6.1627741
H	-23.3283436	-16.2771938	-7.3795724
H	-24.7542413	-17.2538394	-7.8633330
H	-18.9473737	-25.8890174	2.2695005
H	-18.8661737	-26.0718707	0.4749929
H	-20.4255313	-25.6896538	1.2525246
H	-19.4940370	-23.4318102	1.9576176
H	-17.9705918	-23.8178951	1.1363796
H	-20.7640384	-22.0452841	-1.0509050
H	-20.5416656	-21.9238419	0.6639261
H	-20.5941968	-29.5655387	2.0529073
H	-20.5172676	-31.1705853	-0.4429436
H	-20.1409568	-31.6509059	1.1923893
H	-24.4755956	-29.0724349	-3.3352391
H	-24.4006391	-28.6411490	-1.6147705
H	-23.1679985	-29.7282247	-2.3185593
H	-20.4534473	-25.5704935	-2.4541569
H	-23.7426279	-26.7448223	-4.8196068
H	-24.8628350	-24.3028562	-9.1984518
H	-25.4503539	-24.7602810	-7.5706169
H	-24.6501111	-25.9724757	-8.6278336
H	-20.6911634	-22.1634478	-12.8157580
H	-21.3771760	-23.3507094	-11.6099577
H	-19.6042745	-23.2672915	-11.8929677
H	-21.2896764	-22.7193079	-9.2438872
H	-20.3905961	-21.3339570	-8.7321828
H	-14.7485819	-21.9309608	-10.5097950
H	-14.2736812	-21.7578478	-8.8016963
H	-15.0475838	-20.3853603	-9.6502883
H	-17.1977339	-21.5150292	-9.7289217
H	-15.8725035	-23.9408382	-8.3726901
H	-16.4448371	-23.8832184	-10.0605528
H	-17.6105835	-23.7544449	-8.7173759
H	-15.9932406	-21.7908922	-6.9070452
H	-17.7244108	-21.5677081	-7.2999436
H	-16.5700474	-20.2705296	-7.6343502
H	-18.8434627	-28.2219021	-11.8377347
H	-18.0165129	-27.1855484	-13.0248923
H	-19.6969550	-25.8655201	-11.8952229
H	-18.1166419	-25.2622052	-11.3958505
H	-19.8630245	-27.1714211	-9.7015517
H	-19.7490639	-25.4094317	-9.4596963
H	-18.3362928	-26.4332043	-9.1761947
H	-16.8865263	-27.9971668	-10.2514992
H	-16.0772101	-26.8965498	-11.3883006
H	-16.3373231	-28.6030126	-11.8304551
H	-21.1806854	-31.6717408	-10.1382148
H	-20.3064916	-32.3295792	-7.3773850
H	-20.6110695	-33.3911658	-8.7277897
H	-18.2284403	-29.8061486	-5.8303608
H	-17.0753012	-28.4559270	-5.7534374
H	-17.5093561	-29.1856608	-7.3292339
H	-22.0708587	-27.0834467	-7.2453116
H	-18.4930758	-26.0635047	-5.2736544
H	-10.0595822	-19.1386416	-2.3501373
H	-9.1526774	-19.5759453	-0.8616997
H	-9.3891294	-20.8044092	-2.1394744

H	-11.2235232	-20.0272681	0.0068386
H	-14.1492713	-21.5413336	-1.2980998
H	-13.3106258	-20.8637074	0.1395808
H	-11.3105989	-21.1243665	-3.3851025
H	-18.5006034	-17.3424403	-9.4915538
H	-18.3174915	-18.4625311	-8.1165543
H	-19.8395680	-19.2585293	-9.7619342
H	-20.3909303	-16.2010855	-8.2370597
H	-18.8039340	-16.0484423	-7.3649541
H	-20.0371914	-17.2640692	-6.8364081
H	-12.9715841	-21.7681190	-3.2530180
H	-16.4600067	-23.6231292	-4.7579058
H	-16.8099104	-24.2208181	-3.1104637
H	-17.8487714	-21.9505453	-2.5763661
H	-17.7861992	-21.5532328	-4.2951268
H	-21.4860444	-27.6596732	-1.3541884
H	-21.7575247	-23.6404094	4.2089312
H	-23.1791020	-22.7186235	3.6128441
H	-21.5602378	-22.4477610	2.8878531
H	-22.9845008	-26.1450858	0.7619625
H	-24.0220270	-25.8567912	-1.4792895
H	-23.2534796	-21.8732272	1.3807088
H	-24.1774967	-21.5967842	-0.8579776
H	-24.5730978	-24.5629614	-3.2855135
H	-25.1301611	-22.1763607	-6.7577487
H	-23.3306924	-21.1100012	-7.5799654
H	-21.8136560	-19.1380795	-7.4982282
H	-21.6793187	-17.7758104	-5.3912886
H	-23.1565177	-18.3441774	-3.4682729
H	-27.0210303	-22.3296132	-1.7870138
H	-27.4380725	-20.6447211	-2.2512280
H	-26.2709040	-20.9368025	-0.9464582
H	-20.0936987	-22.0237828	-6.5563889
O	-20.1305467	-22.0935451	-3.4643607
H	-27.7127786	-16.4027069	0.7867099
H	-20.5696305	-20.6201286	-6.1785665
H	-20.4439379	-29.0424873	-7.5900141

Supplementary Table 10. Coordinates of the QM cluster model of state 2 of the second reaction sequence, optimized with quintet ferryl.

C	-27.2579693	-17.3879801	0.5119999
O	-27.1594401	-18.2989553	1.3246817
N	-26.7989695	-17.4109811	-0.7359998
C	-25.4809708	-15.4299837	-10.2089881
C	-25.0521136	-14.4717374	-9.1019855
S	-25.5110550	-15.0122323	-7.4105724
C	-24.4029721	-16.4449834	-7.2049920
C	-19.3309766	-25.5119688	1.3099996
C	-19.0431358	-24.0294950	1.0658315
C	-19.2963714	-23.6723879	-0.4019128
O	-19.0094732	-24.4710752	-1.3010244
N	-19.8097745	-22.4554465	-0.6552140
C	-20.7219772	-29.7009687	0.9539997
O	-21.0650546	-28.7687890	0.2322571
N	-20.4449773	-30.9339623	0.5429998
C	-23.7809743	-28.8239689	-2.5189963
C	-22.9496556	-27.6302555	-2.8860972
N	-21.8566079	-27.1959962	-2.1487708
C	-21.3318994	-26.0992077	-2.7493876
N	-22.0383926	-25.7996575	-3.8348222
C	-23.0482075	-26.7396456	-3.9348185
C	-24.6379711	-24.9089710	-8.3089897
C	-23.2818185	-24.5168775	-7.7098888
O	-22.9373000	-25.1304185	-6.6221372
O	-22.5827743	-23.6691042	-8.2699378
C	-20.5389762	-22.6719756	-11.8559857
C	-20.2749041	-21.6500566	-10.7458148
O	-19.6081774	-20.6273375	-10.9815138
N	-20.7163617	-21.9585521	-9.5203985
C	-15.0539838	-21.4899770	-9.5369883
C	-16.4502743	-21.9579450	-9.0913936
C	-16.5771228	-23.4863807	-9.1108900
C	-16.8001393	-21.4008860	-7.7032314
C	-18.1399789	-27.3639693	-11.9339856

C	-18.8156777	-26.1243883	-11.3197461
C	-19.2693802	-26.2939756	-9.8669511
C	-16.7935413	-27.7251000	-11.3044568
C	-20.8929761	-31.3979672	-9.0979914
O	-20.8250852	-30.2205308	-8.7620510
N	-20.5799772	-32.4509679	-8.3489907
C	-17.9129800	-28.8779689	-6.3399922
C	-19.0210231	-27.8929907	-6.4313472
N	-20.2056162	-28.1865206	-7.0756196
C	-21.0337102	-27.1393376	-6.9471261
N	-20.4467317	-26.1754785	-6.2444237
C	-19.1843420	-26.6286133	-5.9142733
C	-9.8599882	-19.9459764	-1.6139985
N	-11.1323053	-20.3718103	-1.0070262
C	-12.2180992	-20.8840466	-1.6492749
N	-13.3652644	-21.0502296	-0.9878369
N	-12.1778737	-21.2449663	-2.9351505
C	-19.0201846	-17.8082619	-8.6483009
O	-20.0859903	-18.6314677	-9.0753397
C	-19.5839765	-16.7449823	-7.7059918
Fe	-21.4259955	-24.5399601	-5.4805991
O	-14.5280521	-22.5496910	-3.9992558
C	-15.5562449	-22.5697446	-3.2702928
O	-15.6491454	-22.0123779	-2.1377446
C	-16.8159703	-23.3434002	-3.7620135
C	-18.0260217	-22.4106717	-3.9440047
C	-19.4512893	-22.9785548	-4.0322545
O	-19.6731377	-23.9935543	-4.8286799
C	-20.4077950	-19.7395133	-3.2825604
O	-19.7673465	-19.6392822	-4.2503530
O	-21.0371161	-19.7660109	-2.3057899
C	-21.7846226	-21.2686578	3.4315135
O	-21.9815462	-22.5357876	2.8254495
C	-22.4544166	-22.5949502	1.5543738
C	-22.6460257	-23.8831608	1.0242036
C	-23.0844186	-24.0432015	-0.2831750
C	-22.7199786	-21.4735388	0.7505822
C	-23.1771794	-21.6498738	-0.5574218
C	-23.3829820	-22.9304041	-1.1014831
C	-23.8588716	-23.1693922	-2.4653005
C	-24.6323499	-22.3882392	-3.2447600
C	-24.9729384	-22.8515105	-4.6305299
O	-25.3535288	-23.9839017	-4.8848990
N	-24.8515975	-21.8984569	-5.6198003
C	-24.0500475	-20.7427875	-5.5731503
C	-23.2462002	-20.4648546	-6.6946696
C	-22.4277827	-19.3403482	-6.7314372
C	-22.4026759	-18.4656463	-5.6356557
C	-23.2142432	-18.7141136	-4.5360179
C	-24.0427309	-19.8501295	-4.4768082
C	-24.9405492	-19.9465149	-3.2860705
O	-25.3662155	-18.9256345	-2.7352782
N	-25.2612519	-21.1818277	-2.8026341
C	-26.2183276	-21.2359352	-1.6924133
O	-22.1168940	-23.0783237	-5.2554175
O	-20.1665540	-21.4452706	-6.7164366
H	-26.9280677	-16.6067140	-1.3400501
H	-26.3064863	-18.2097207	-1.1454481
H	-25.2097607	-15.0228672	-11.1983450
H	-26.5702307	-15.5936490	-10.1871823
H	-24.9875573	-16.4099363	-10.1089161
H	-25.5401253	-13.4907475	-9.2219899
H	-23.9627987	-14.2961344	-9.1272388
H	-24.5641229	-16.8316764	-6.1902691
H	-23.3469373	-16.1493036	-7.3034150
H	-24.6218830	-17.2532743	-7.9187060
H	-18.9519597	-25.8683374	2.2787487
H	-18.8661677	-26.0718637	0.4749929
H	-20.4255253	-25.6896458	1.2525246
H	-19.6361814	-23.3843529	1.7343016
H	-17.9782082	-23.8073587	1.2605019
H	-20.0788742	-22.2452960	-1.6250506
H	-20.1801748	-21.8815841	0.0901346
H	-20.6157981	-29.5716643	2.0561521
H	-20.4955885	-31.1642110	-0.4457773
H	-20.1598302	-31.6562917	1.1949097
H	-24.4683770	-29.0584562	-3.3446767

H	-24.4006321	-28.6411420	-1.6147705
H	-23.1679925	-29.7282157	-2.3185583
H	-20.4631162	-25.5480942	-2.3786513
H	-23.7667409	-26.6926305	-4.7486197
H	-24.8266895	-24.2832699	-9.1917899
H	-25.4503469	-24.7602740	-7.5706149
H	-24.6501031	-25.9724677	-8.6278306
H	-20.6895203	-22.1663134	-12.8179580
H	-21.3771700	-23.3507034	-11.6099547
H	-19.6042695	-23.2672855	-11.8929637
H	-21.3134642	-22.7586773	-9.3132901
H	-20.4407611	-21.4072501	-8.7009679
H	-14.7485779	-21.9309558	-10.5097920
H	-14.2875972	-21.7641845	-8.7902790
H	-15.0475798	-20.3853553	-9.6502863
H	-17.1884357	-21.5502079	-9.8086495
H	-15.8702184	-23.9488681	-8.3984059
H	-16.3623956	-23.8991213	-10.1116989
H	-17.5930555	-23.8041713	-8.8246855
H	-16.1109283	-21.7937980	-6.9329389
H	-17.8279426	-21.6684737	-7.4108162
H	-16.7236589	-20.2997470	-7.6866498
H	-18.8434567	-28.2218951	-11.8377317
H	-18.0165089	-27.1855414	-13.0248883
H	-19.6921003	-25.8612535	-11.9383470
H	-18.1262117	-25.2624569	-11.3900580
H	-19.9219948	-27.1765581	-9.7556855
H	-19.8321778	-25.4140874	-9.5144173
H	-18.4203705	-26.4267864	-9.1773054
H	-16.8994565	-27.9687711	-10.2341572
H	-16.0829916	-26.8839303	-11.3809405
H	-16.3358973	-28.5976633	-11.7992507
H	-21.2438030	-31.6770770	-10.1172991
H	-20.2485282	-32.3260817	-7.3960017
H	-20.6658924	-33.3945454	-8.7104246
H	-18.2440189	-29.7989368	-5.8285382
H	-17.0752972	-28.4559190	-5.7534364
H	-17.5093511	-29.1856528	-7.3292319
H	-22.0440419	-27.0977718	-7.3388163
H	-18.5096762	-26.0115773	-5.3277774
H	-10.0595792	-19.1386356	-2.3501363
H	-9.1911301	-19.5876282	-0.8237522
H	-9.3891274	-20.8044032	-2.1394734
H	-11.2975078	-20.0693389	-0.0542673
H	-14.2430522	-21.4218772	-1.4696213
H	-13.4345009	-20.7511987	-0.0225902
H	-11.3227476	-21.1800507	-3.4707263
H	-18.5213191	-17.3163187	-9.5101514
H	-18.2370128	-18.3953773	-8.1234052
H	-19.7749740	-19.2976576	-9.7307228
H	-20.3909243	-16.2010815	-8.2370577
H	-18.8138253	-16.0396469	-7.3594483
H	-20.0371854	-17.2640642	-6.8364061
H	-13.0211310	-21.7017450	-3.3656433
H	-16.5762066	-23.8774027	-4.6934463
H	-17.0664244	-24.0779358	-2.9789227
H	-18.0428675	-21.7148031	-3.0940259
H	-17.9042007	-21.8082154	-4.8615063
H	-21.4932432	-27.6623867	-1.3095418
H	-21.4271686	-21.4639898	4.4515205
H	-22.7245228	-20.6915559	3.4806226
H	-21.0259669	-20.6732238	2.8926500
H	-22.4322447	-24.7435918	1.6607942
H	-23.2069114	-25.0521601	-0.6856050
H	-22.5644442	-20.4608155	1.1228360
H	-23.3364414	-20.7648677	-1.1724546
H	-23.5557835	-24.1151743	-2.9181909
H	-24.9854894	-22.2920382	-6.5477910
H	-23.2286788	-21.1832665	-7.5180651
H	-21.7828740	-19.1581771	-7.5959084
H	-21.7625503	-17.5827003	-5.6505606
H	-23.2346692	-18.0254210	-3.6894554
H	-26.4705951	-22.2845814	-1.4959217
H	-27.1274249	-20.6783024	-1.9615758
H	-25.8063601	-20.7855348	-0.7753324
H	-20.9029057	-21.8918513	-6.2544506
O	-20.3439152	-22.4170075	-3.3958692

H	-27.7641066	-16.4227381	0.7677354
H	-19.9797955	-20.6809623	-6.1542942
H	-20.4112450	-29.0429111	-7.6153042

Supplementary Table 11. Coordinates of the QM cluster model of state 3 of the second reaction sequence, optimized with quintet ferryl.

C	-27.2579610	-17.3879760	0.5120000
O	-27.1712439	-18.2996904	1.3223816
N	-26.7989620	-17.4109750	-0.7360000
C	-25.4809630	-15.4299790	-10.2089850
C	-24.9860126	-14.4761552	-9.1256002
S	-25.4413644	-14.9619413	-7.4163226
C	-24.4029640	-16.4449790	-7.2049900
C	-19.3309710	-25.5119610	1.3100000
C	-19.0097898	-24.0295230	1.0756610
C	-19.2911430	-23.6290420	-0.3766589
O	-19.0418274	-24.4107581	-1.2988242
N	-19.8008363	-22.4013439	-0.5969132
C	-20.7219720	-29.7009600	0.9540000
O	-21.1255789	-28.7873433	0.2425665
N	-20.4449710	-30.9339540	0.5430000
C	-23.7809660	-28.8239610	-2.5189950
C	-22.9245123	-27.6508652	-2.8724830
N	-21.9316488	-27.1513069	-2.0490361
C	-21.3689945	-26.0757232	-2.6542217
N	-21.9432031	-25.8593824	-3.8305743
C	-22.9061155	-26.8381472	-3.9851001
C	-24.6379640	-24.9089640	-8.3089880
C	-23.2463184	-24.5591090	-7.7716148
O	-23.0889918	-24.6096863	-6.4905861
O	-22.3529150	-24.2839166	-8.5821503
C	-20.5389710	-22.6719700	-11.8559830
C	-20.3118683	-21.7225670	-10.6726235
O	-19.7012604	-20.6469038	-10.8237088
N	-20.7372323	-22.1568537	-9.4786954
C	-15.0539800	-21.4899710	-9.5369850
C	-16.4389646	-21.9606121	-9.0639795
C	-16.5689311	-23.4890496	-9.0838335
C	-16.7470608	-21.4054325	-7.6658729
C	-18.1399730	-27.3639620	-11.9339830
C	-18.8226402	-26.1266671	-11.3222792
C	-19.2901104	-26.3068745	-9.8760499
C	-16.7954390	-27.7209005	-11.2989865
C	-20.8929710	-31.3979570	-9.0979880
O	-20.8870630	-30.2250776	-8.7427145
N	-20.5799710	-32.4509620	-8.3489880
C	-17.9129730	-28.8779620	-6.3399900
C	-19.0056901	-27.8702450	-6.4622154
N	-20.1815287	-28.1423141	-7.1280754
C	-20.9701893	-27.0491220	-7.0599398
N	-20.3743688	-26.0868494	-6.3770497
C	-19.1439912	-26.5786605	-5.9957821
C	-9.8599850	-19.9459710	-1.6139990
N	-11.1192173	-20.3851721	-1.0048207
C	-12.2396397	-20.7785556	-1.7055201
N	-13.4283167	-20.9075873	-1.1776195
N	-12.0836311	-21.0235893	-3.0436610
C	-18.9644741	-17.8182091	-8.6003368
O	-19.8908938	-18.8888036	-8.7305377
C	-19.5839710	-16.7449780	-7.7059900
Fe	-21.4048392	-24.4958665	-5.4058069
O	-14.7153408	-22.3295542	-4.1252851
C	-15.6844112	-22.4126259	-3.3766373
O	-15.6913115	-21.9454473	-2.1433893
C	-17.0005018	-23.0708436	-3.7793708
C	-18.1596181	-22.0622874	-3.8554255
C	-19.5615827	-22.6561132	-4.0160249
O	-19.7124788	-23.6025902	-4.8691711
C	-20.6071379	-19.5871350	-2.9704701
O	-20.0011569	-19.3543127	-3.9369938
O	-21.2170822	-19.7322898	-1.9893818
C	-22.6019144	-24.1414989	2.9881712
O	-23.3786742	-24.9965081	2.1688936
C	-23.5490213	-24.6840940	0.8572891

C	-24.4619574	-25.4822976	0.1442189
C	-24.6998049	-25.2327837	-1.2034306
C	-22.8712504	-23.6478617	0.1948263
C	-23.1144734	-23.4085025	-1.1611128
C	-24.0415001	-24.1836316	-1.8655110
C	-24.2956873	-23.9562183	-3.3128309
C	-24.8600644	-22.7291630	-3.9060308
C	-25.5649014	-22.8593310	-5.2457586
O	-26.3469903	-23.7592754	-5.5018465
N	-25.2422463	-21.8706683	-6.1288384
C	-24.3102396	-20.8297349	-5.9454802
C	-23.5367671	-20.4610353	-7.0606430
C	-22.6329880	-19.4049644	-6.9925433
C	-22.4855861	-18.6965671	-5.7941602
C	-23.2527121	-19.0465470	-4.6888440
C	-24.1634653	-20.1167082	-4.7290520
C	-24.9564593	-20.3311273	-3.4688773
O	-25.2603055	-19.3830546	-2.7465001
N	-25.3017027	-21.6147317	-3.1284694
C	-26.1490124	-21.8166180	-1.9507527
O	-23.4656728	-22.9977587	-3.9761104
O	-19.9391919	-20.9550074	-6.8796311
H	-26.9108928	-16.6087714	-1.3454411
H	-26.3171719	-18.2280352	-1.1155035
H	-25.2059117	-15.0531951	-11.2091448
H	-26.5765747	-15.5377731	-10.1675576
H	-25.0361123	-16.4320476	-10.0999063
H	-25.4265559	-13.4741578	-9.2551043
H	-23.8898876	-14.3554019	-9.1713001
H	-24.5508063	-16.7995808	-6.1758564
H	-23.3363858	-16.2070097	-7.3425971
H	-24.6848079	-17.2577347	-7.8908644
H	-18.9527136	-25.8752317	2.2763869
H	-18.8661610	-26.0718560	0.4749930
H	-20.4255200	-25.6896380	1.2525250
H	-19.5498995	-23.3688933	1.7742847
H	-17.9329319	-23.8480925	1.2444597
H	-20.0685696	-22.1516727	-1.5610018
H	-20.0174171	-21.7707677	0.1641034
H	-20.5517231	-29.5555299	2.0466274
H	-20.5519511	-31.1788564	-0.4377314
H	-20.1090756	-31.6422428	1.1860082
H	-24.4613156	-29.0526510	-3.3520292
H	-24.4006250	-28.6411340	-1.6147710
H	-23.1679860	-29.7282090	-2.3185570
H	-20.5557321	-25.4859168	-2.2262589
H	-23.5241975	-26.8816028	-4.8794554
H	-24.8317730	-24.3090917	-9.2108762
H	-25.4503380	-24.7602670	-7.5706130
H	-24.6500970	-25.9724600	-8.6278290
H	-20.6875078	-22.1227999	-12.7930009
H	-21.3771650	-23.3506970	-11.6099520
H	-19.6042630	-23.2672790	-11.8929600
H	-21.2641474	-23.0239719	-9.3366456
H	-20.4859655	-21.6627624	-8.6150450
H	-14.7485740	-21.9309500	-10.5097890
H	-14.2777413	-21.7587825	-8.7981523
H	-15.0475760	-20.3853490	-9.6502850
H	-17.1961039	-21.5510961	-9.7603747
H	-15.8477729	-23.9535688	-8.3873357
H	-16.3762056	-23.9007638	-10.0896731
H	-17.5793221	-23.8055104	-8.7768372
H	-16.0271160	-21.7898072	-6.9198920
H	-17.7612362	-21.6722365	-7.3342244
H	-16.6785141	-20.3031685	-7.6541599
H	-18.8434510	-28.2218880	-11.8377280
H	-18.0165020	-27.1855340	-13.0248840
H	-19.6937694	-25.8644858	-11.9489834
H	-18.1348642	-25.2623124	-11.3840547
H	-19.9172800	-27.2086852	-9.7767646
H	-19.8871833	-25.4502899	-9.5253170
H	-18.4477514	-26.4198774	-9.1747669
H	-16.9064688	-27.9611151	-10.2284338
H	-16.0865299	-26.8781883	-11.3744716
H	-16.3333851	-28.5938825	-11.7891441
H	-21.1834647	-31.6753659	-10.1370785
H	-20.3010762	-32.3289968	-7.3789459

H	-20.6103990	-33.3908229	-8.7282647
H	-18.2593197	-29.7924744	-5.8277480
H	-17.0752910	-28.4559120	-5.7534360
H	-17.5093480	-29.1856460	-7.3292300
H	-21.9571683	-26.9874429	-7.5100494
H	-18.4599110	-25.9704098	-5.4080972
H	-10.0595760	-19.1386300	-2.3501350
H	-9.1801784	-19.5883955	-0.8311198
H	-9.3891260	-20.8043980	-2.1394720
H	-11.2895968	-20.0982594	-0.0484502
H	-14.7801110	-21.5334718	-1.8546996
H	-13.4583683	-20.6078724	-0.2027419
H	-11.1775090	-21.3168259	-3.3888812
H	-18.7092930	-17.3994384	-9.5922931
H	-18.0189654	-18.1815986	-8.1497718
H	-19.6968784	-19.4659064	-9.5156406
H	-20.3909190	-16.2010780	-8.2370560
H	-18.8343597	-16.0204796	-7.3534595
H	-20.0371800	-17.2640610	-6.8364040
H	-12.8901416	-21.4614201	-3.4990635
H	-16.8601123	-23.5738644	-4.7454420
H	-17.2502909	-23.8220377	-3.0120183
H	-18.1629373	-21.4422171	-2.9486535
H	-18.0244505	-21.3873466	-4.7186789
H	-21.6446456	-27.5692751	-1.1585260
H	-22.6755496	-24.5358195	4.0107755
H	-22.9829898	-23.1046755	2.9713485
H	-21.5408382	-24.1365905	2.6862773
H	-24.9673658	-26.2943215	0.6717069
H	-25.4013273	-25.8657617	-1.7535230
H	-22.1315509	-23.0398786	0.7124899
H	-22.5553328	-22.6359909	-1.6934810
H	-24.4933854	-24.8592188	-3.8992944
H	-25.5948466	-22.0136393	-7.0725441
H	-23.6351345	-21.0354615	-7.9854632
H	-22.0222576	-19.1463149	-7.8597142
H	-21.7774201	-17.8701494	-5.7241772
H	-23.1716624	-18.4893815	-3.7537944
H	-26.5576217	-22.8334091	-1.9750318
H	-26.9714839	-21.0880473	-1.9617874
H	-25.5749021	-21.6837790	-1.0211739
H	-20.5195379	-20.7925388	-6.1232811
O	-20.4969328	-22.1686237	-3.3553609
H	-27.7539002	-16.4171731	0.7665796
H	-19.8758540	-20.0955657	-7.3510299
H	-20.4067244	-29.0054211	-7.6418720

Supplementary References

[1] Bräuer, A., Beck, P., Hintermann, L. & Groll, M. Structure of the Dioxygenase AsqJ: Mechanistic Insights into a One-Pot Multistep Quinolone Antibiotic Biosynthesis. *Angew. Chem. Int. Ed.* **55**, 422–426 (2016); *Angew. Chem.* **128**, 432–436 (2016).
