# Supplementary material for: Catalytic mechanism and molecular engineering of quinolone biosynthesis in dioxygenase AsqJ
Source: Nat Commun. 2018 Mar 21;9:1168. doi: 10.1038/s41467-018-03442-2 (PMC5862883; doi:10.1038/s41467-018-03442-2)
Supplement: Supplementary file 2 — Description of Additional Supplementary Files(PDF 165 kb) [file 41467_2018_3442_MOESM2_ESM.pdf]

Description of Additional Supplementary Files

File Name: Supplementary Movie 1
Description: Animation of the first part of the catalytic cycle based on quantum chemical DFT calculations.

File Name: Supplementary Movie 2
Description: Animation of the second part of catalytic cycle based on quantum chemical DFT calculations.
