# Supplementary figures and images for: Catalytic mechanism and molecular engineering of quinolone biosynthesis in dioxygenase AsqJ
Source: Nat Commun. 2018 Mar 21;9:1168. doi: 10.1038/s41467-018-03442-2 (PMC5862883; doi:10.1038/s41467-018-03442-2)

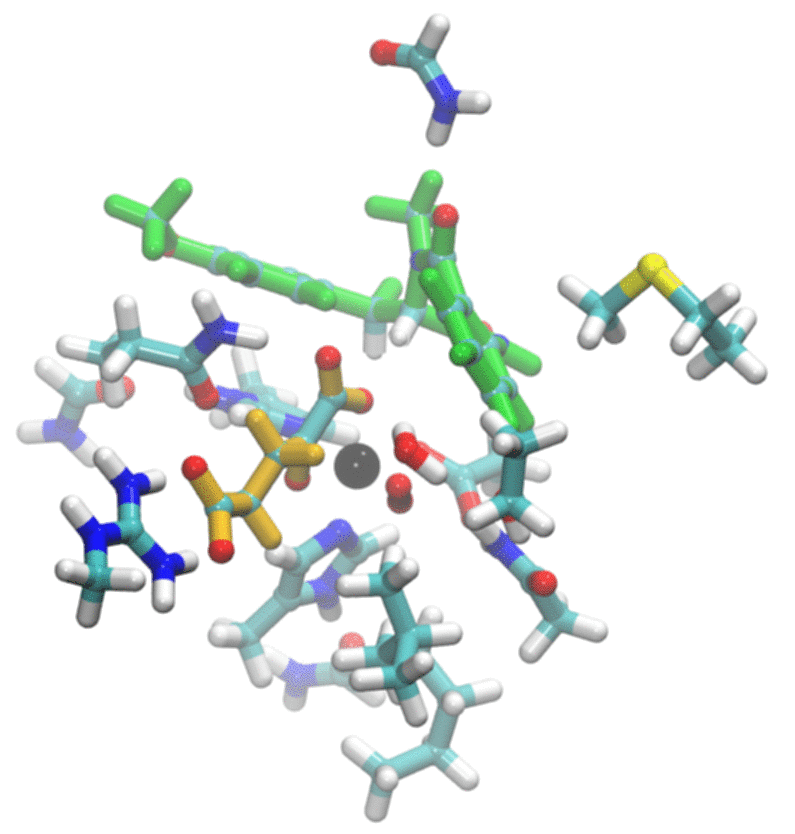

Supplement: Supplementary file 3 — Supplementary Movie 1(GIF 5879 kb) [file 41467_2018_3442_MOESM3_ESM.gif]

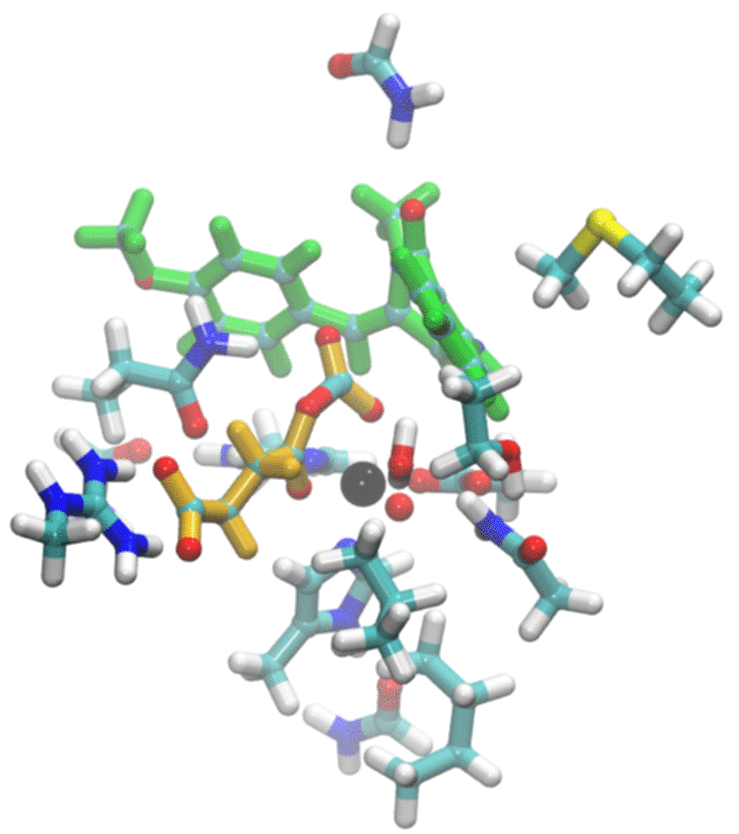

Supplement: Supplementary file 4 — Supplementary Movie 2(GIF 2780 kb) [file 41467_2018_3442_MOESM4_ESM.gif]
